# Natural products targeting glycolytic signaling pathways-an updated review on anti-cancer therapy

**DOI:** 10.3389/fphar.2022.1035882

**Published:** 2022-10-20

**Authors:** Yuting Cui, Chuang Li, Feng Sang, Weiling Cao, Zhuo Qin, Peng Zhang

**Affiliations:** ^1^ School of Life Sciences and Medicine, Shandong University of Technology, Zibo, Shandong, China; ^2^ Department of Pharmacy, Shenzhen Luohu People’s Hospital, Shenzhen, Guangdong, China

**Keywords:** glycolysis, natural compounds, anti-cancer, hexokinase (HK), phosphofructokinase-1 (PFK-1), pyruvate kinase (PK)

## Abstract

Glycolysis is a complex metabolic process that occurs to convert glucose into pyruvate to produce energy for living cells. Normal cells oxidized pyruvate into adenosine triphosphate and carbon dioxide in the presence of oxygen in mitochondria while cancer cells preferentially metabolize pyruvate to lactate even in the presence of oxygen in order to maintain a slightly acidic micro-environment of PH 6.5 and 6.9, which is beneficial for cancer cell growth and metastasis. Therefore targeting glycolytic signaling pathways provided new strategy for anti-cancer therapy. Natural products are important sources for the treatment of diseases with a variety of pharmacologic activities. Accumulated studies suggested that natural products exhibited remarkable anti-cancer properties both *in vitro* and *in vivo*. Plenty of studies suggested natural products like flavonoids, terpenoids and quinones played anti-cancer properties *via* inhibiting glucose metabolism targets in glycolytic pathways. This study provided an updated overview of natural products controlling glycolytic pathways, which also provide insight into druggable mediators discovery targeting cancer glucose metabolism.

## Introduction

### Glycolysis in tumor environment

In most organisms, glycolysis is the process through which glucose is prepared for aerobic oxidation. Six carbon glucose molecules are broken into two molecules of three carbon pyruvate in more than ten steps of enzyme catalyzed reaction in this process, while two molecules of adenosine diphosphate (ADP) and inorganic phosphate (PI) are joined to generate two molecules of adenosine triphosphate (ATP). Normal cells degrade pyruvate to carbon dioxide and water in the presence of oxygen, but will convert it to lactic acid, ethanol, or acetic acid in the absence of oxygen. However, in cancer cells, in order to meet the increased energy needs brought on by tumor cell multiplication, their metabolism must be reprogrammed. Therefore, even under aerobic conditions, ATP is primarily created by glycolysis, a process also known as the Warburg Effect to meet the high energy requirement.

The basic way to regulate glycolysis is to change the activity of enzymes . Key glycolysis enzymes include hexokinase (HK), phosphofructokinase-1 (PFK-1) and pyruvate kinase (PK). Their activities have a direct impact on the speed and direction of the entire metabolic pathway. HKs are the first glycolysis rate-limiting enzymes that irreversibly phosphorylate glucose to glucose-6-phosphate. Four distinct HK isoforms have been discovered in mammals. They are scattered throughout the cell in various locations. HK2 is situated on the outer mitochondrial membrane, which has the advantage of avoiding product inhibition and improving apparent binding with ATP. It also suppresses the release of cytochrome *c* by interacting withvoltage-dependent anion channels (VDAC), hence increasing aerobic glycolysis in tumor cells and reducing apoptosis (Pastorino et al., 2002; Wilson, 2003; Pedersen, 2008). Recently, it has been discovered that high HK2 expression is associated with a poor prognosis and endpoint of cancer in several tumors, indicating that HK2 is a possible therapeutic target for cancer (Ros and Schulze, 2013; Katagiri et al., 2017; Zhou et al., 2019). PFK-1 is the second rate-limiting enzyme in glycolysis and its activity is mainly regulated by Phosphofructokinase-2/fructose-2,6-diphosphate 3 (PFKFB3), which catalyzes the formation of fructose 2,6-diphosphate from fructose 6-phosphate rather than directly participate in the catalytic process of glycolysis. Since fructose 2,6-diphosphate is an allosteric activator of PFK-1 that can boost its catalytic activity considerably, PFKFB3 is necessary for the regulation of glycolysis and is an important therapeutic target for cancer. PK is the third rate-limiting enzyme in glycolysis. It catalyzes the last process in the conversion of glucose to pyruvate and simultaneously transfers the phosphate group from phosphoenolpyruvate to ADP to generate pyruvate (Vander Heiden et al., 2009). PK has four isoenzymes: PKL, PKR, PKM1 and PKM2. Each of these isoenzymes is specific to a certain type of tissue (Chaneton and Gottlieb, 2012). Among them, PKM1 is found to be up-regulated in tissues that require a large amount of energy supply, such as heart, brain and muscle. PKM2 is expressed in all proliferating cells, especially in tumor and embryonic tissues (Zahra et al., 2020). PKM2 mainly includes two configurations, namely dimer and tetramer, and is mainly presented as dimer in cancer cells, where it facilitates the production of nucleic acid and protein as well as maintaining the aerobic glycolytic pathway (Liu et al., 2014; Yoon et al., 2018), as a result, PKM2 is expected to be a therapeutic target for various cancers. Lactate dehydrogenase (LDH) is a tetrameric enzyme, which catalyzes the last step of glycolysis and is responsible for the mutual conversion of pyruvate and lactic acid. LDH is a tetramer composed of M (LDHA encoded) and H (encoded by LDHB) subunits, and can be classified into five subtypes (LDH1 to LDH5) based on its subunit composition. LDHA is responsible for converting pyruvate into lactic acid for glycolysis and mainly exists in tissues with frequent oxygen deficiency, such as muscle. LDHB is responsible for converting lactic acid into pyruvate which goes into tricarboxylic acid cycle and mainly exists in oxygen-rich tissues such as the heart (Markert, 1963; Sutendra and Michelakis, 2013). An increase in the proportion of type M subunit and an increase in LDH5 are frequently observed in cases of hypoxia, anemia, etc. and in a wide range of malignant tumors (Kolev et al., 2008; Kotyza et al., 2009). Recent studies have shown that LDHA is over-expressed in various types of cancers, such as gastric cancer, breast cancer and pancreatic cancer (Kolev et al., 2008; Wang et al., 2012a; Cheng et al., 2021).

The regulation of glycolysis is also regulated by glucose transporters (GLUT). GLUT is a transporter family that responsible for transporting extracellular glucose into cells. Hypoxic tumor microenvironment causes high expression of GLUT1 in most tumors, which makes it easier for tumor cells to take in glucose and serves as the foundation for the Warburg Effect of tumor cells. At present, there are 14 members in the GLUT family, but only GLUT1-5 have been studied most deeply. GLUT1 is the most widely distributed glucose transporter, and its expression is mainly regulated by HIF-1α. Therefore, GLUT and HIF-1α have become potential targets for the treatment of tumorigenesis and development.

Transport of lactic acid out of cells is dependent on monocarboxylic acid transporters. The MCT family, which currently consists of 14 members, is a crucial transmembrane transporter on the membrane of mammalian cells, with specific substrate needs and tissue distribution preferences for each subtype (Halestrap, 2012). It regulates the transmembrane transport of monocarboxylic acid compounds such as pyruvic acid, lactic acid, ketone bodies and short chain fatty acids. In addition, it is involved in drug administration, nutrition absorption, metabolic dynamic equilibrium, and other biological activities (Pinheiro et al., 2012). Glycolysis is specifically rapid and this way of supplying energy may lead to an increase in the generation of lactic acid in cancer cells. The accumulation of lactic acid will have an impact on tumor cell development and proliferation, and may even cause tumor cell death if the transport system is unable to handle the lactic acid. According to previous studies, MCT1 and MCT4 are the primary transporters for lactic acid expulsion in tumor cells (Tai et al., 2005; Morais-Santos et al., 2014; Azevedo et al., 2015; Martel et al., 2016; Wang et al., 2019; Ruzzolini et al., 2020; Soni et al., 2020), as a result, MCT has the potential to be used as a target for tumor-targeted therapy.

### Natural products targeting glycolysis for cancer therapy

**FIGURE 1:**
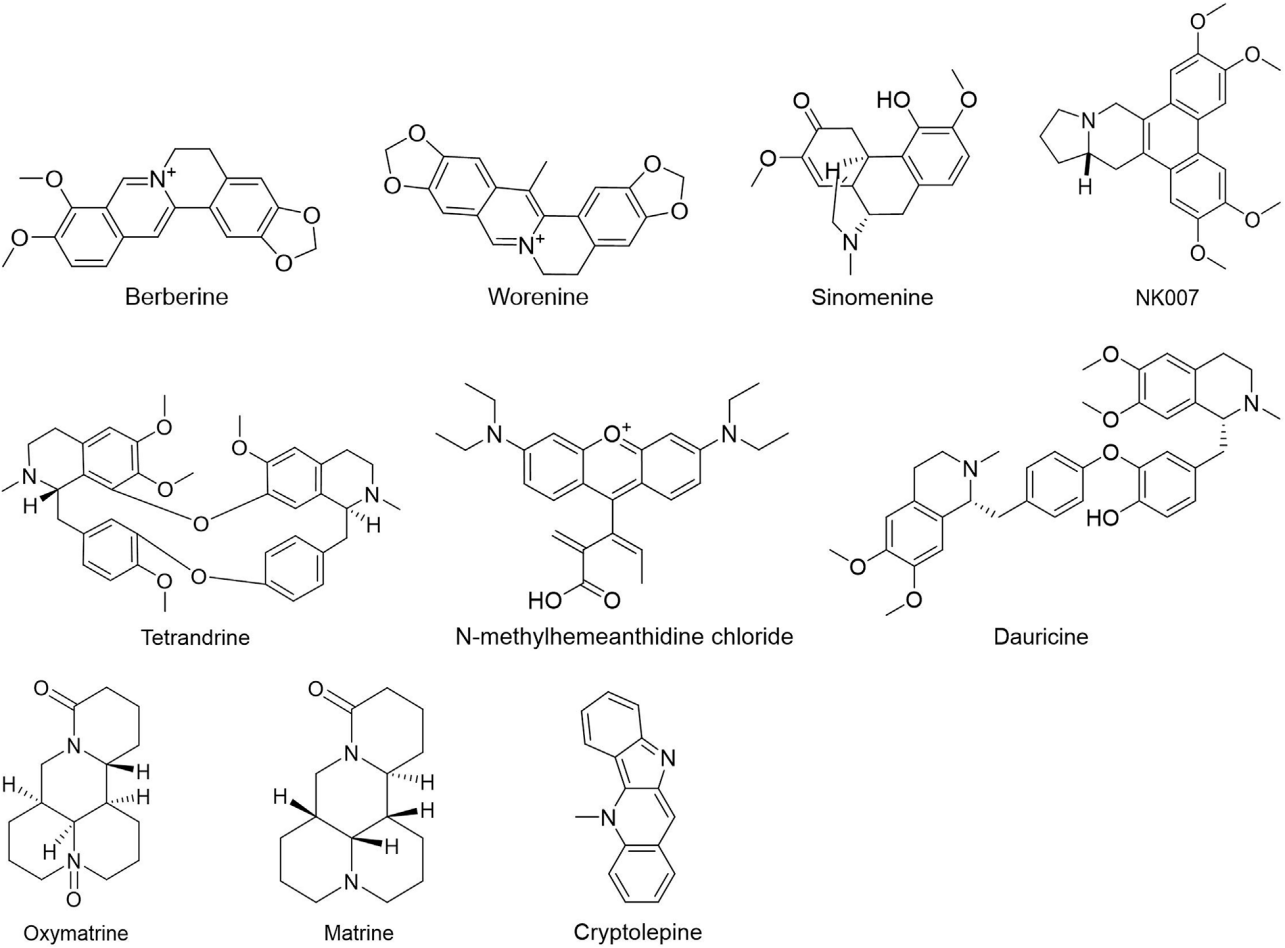
Alkaloids.

**FIGURE 2:**
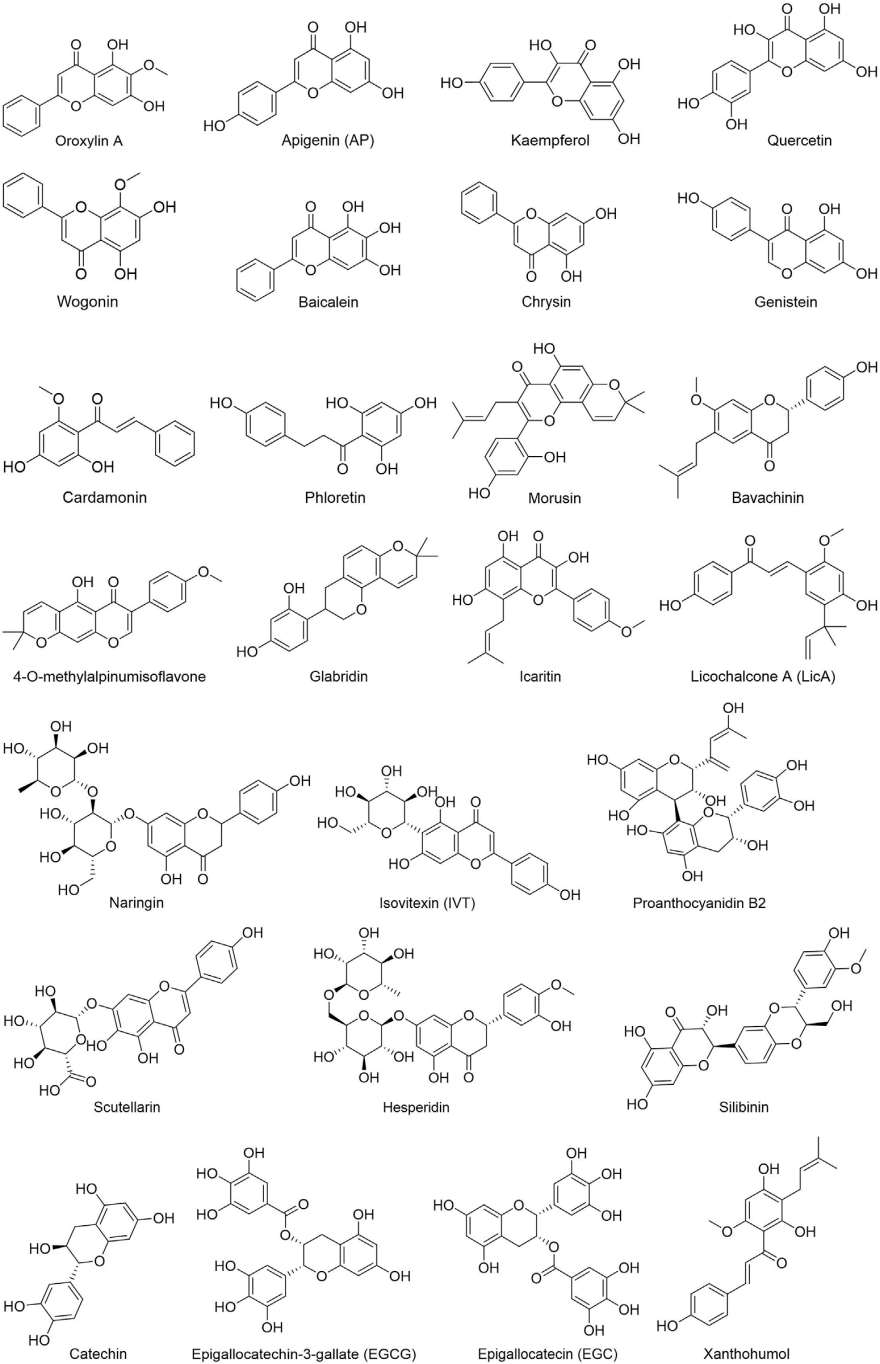
Flavonoids.

**FIGURE 3:**
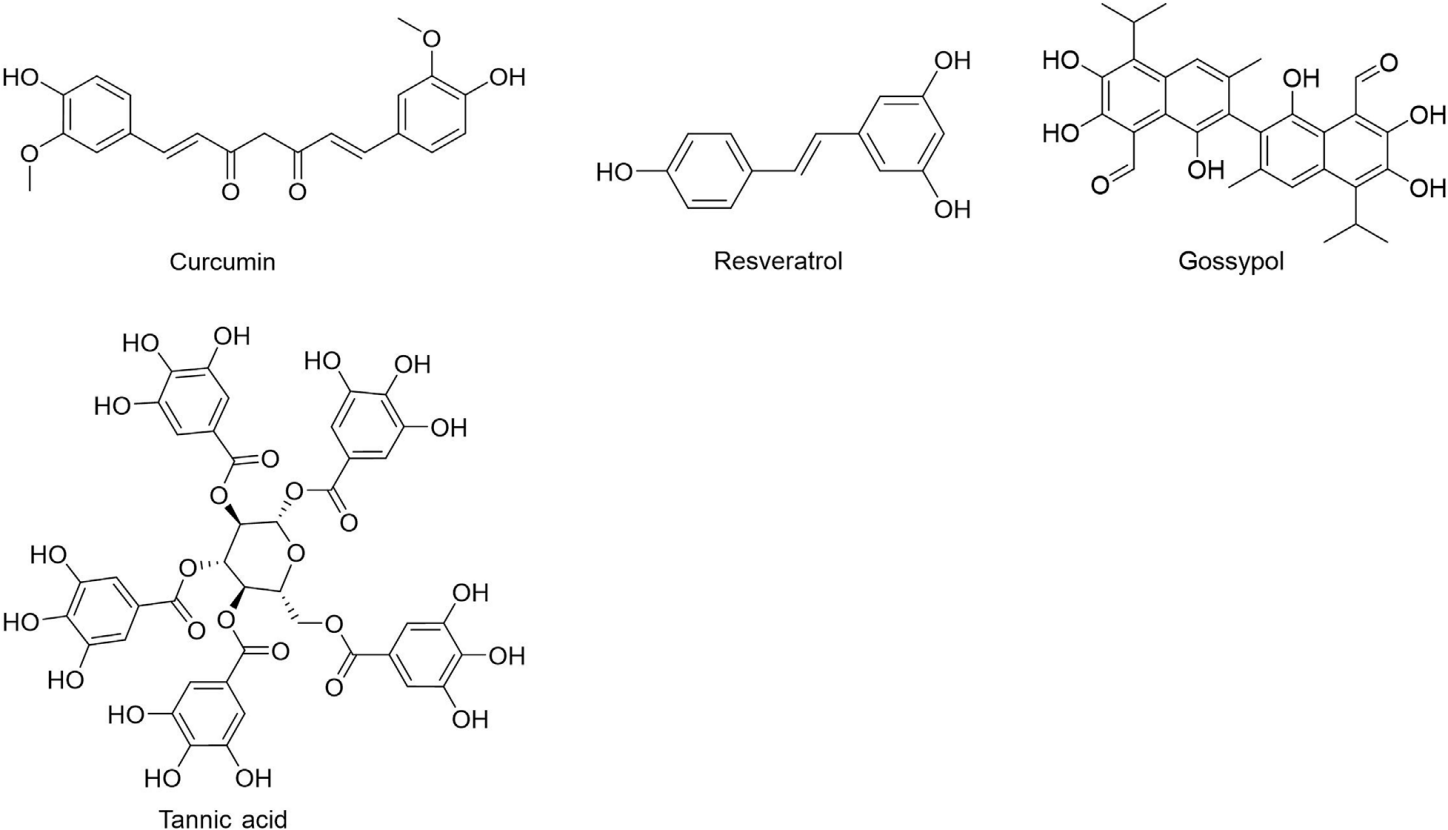
Non-flavonoid phenolic compounds.

**FIGURE 4:**
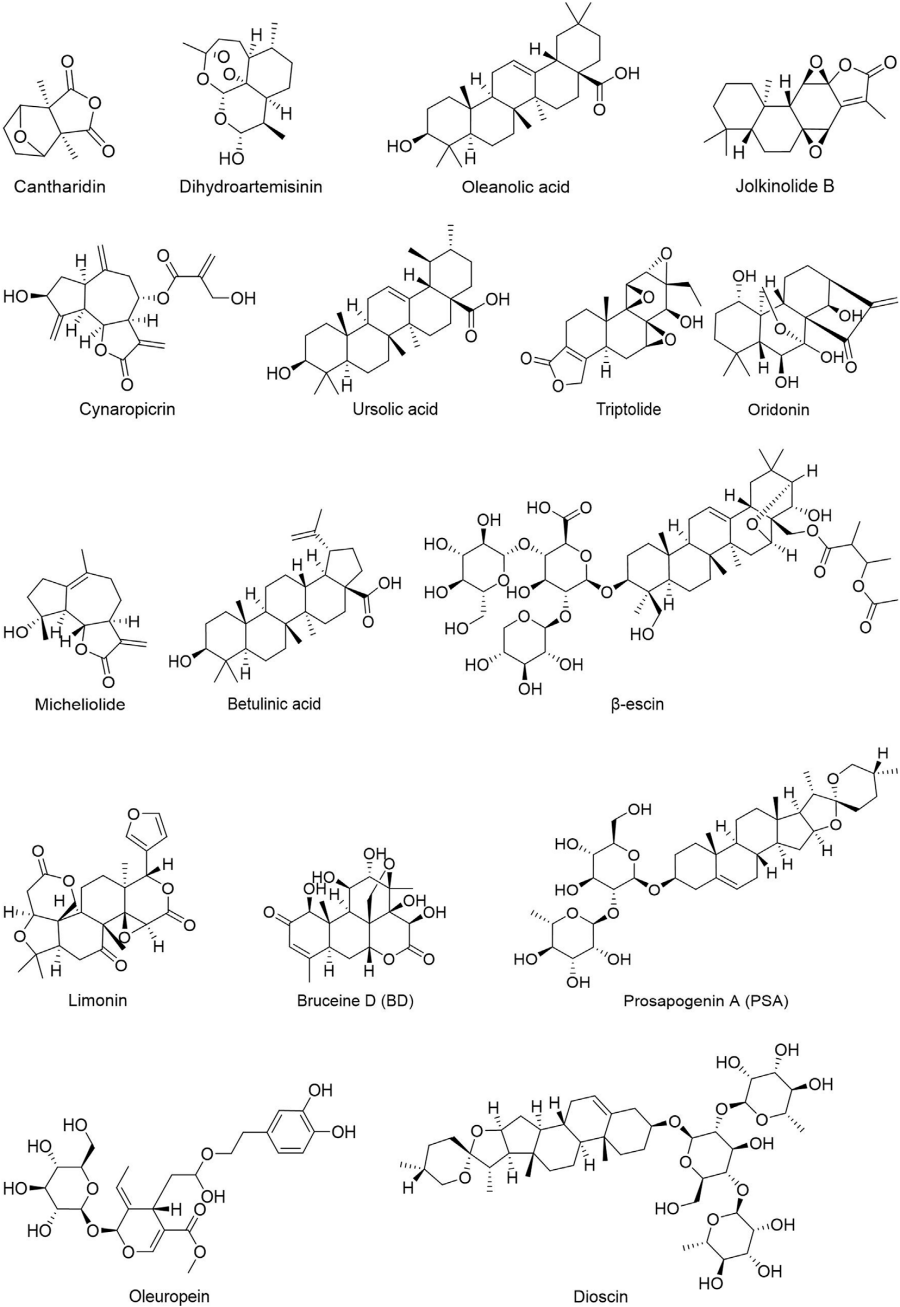
Terpenoids.

**FIGURE 5:**
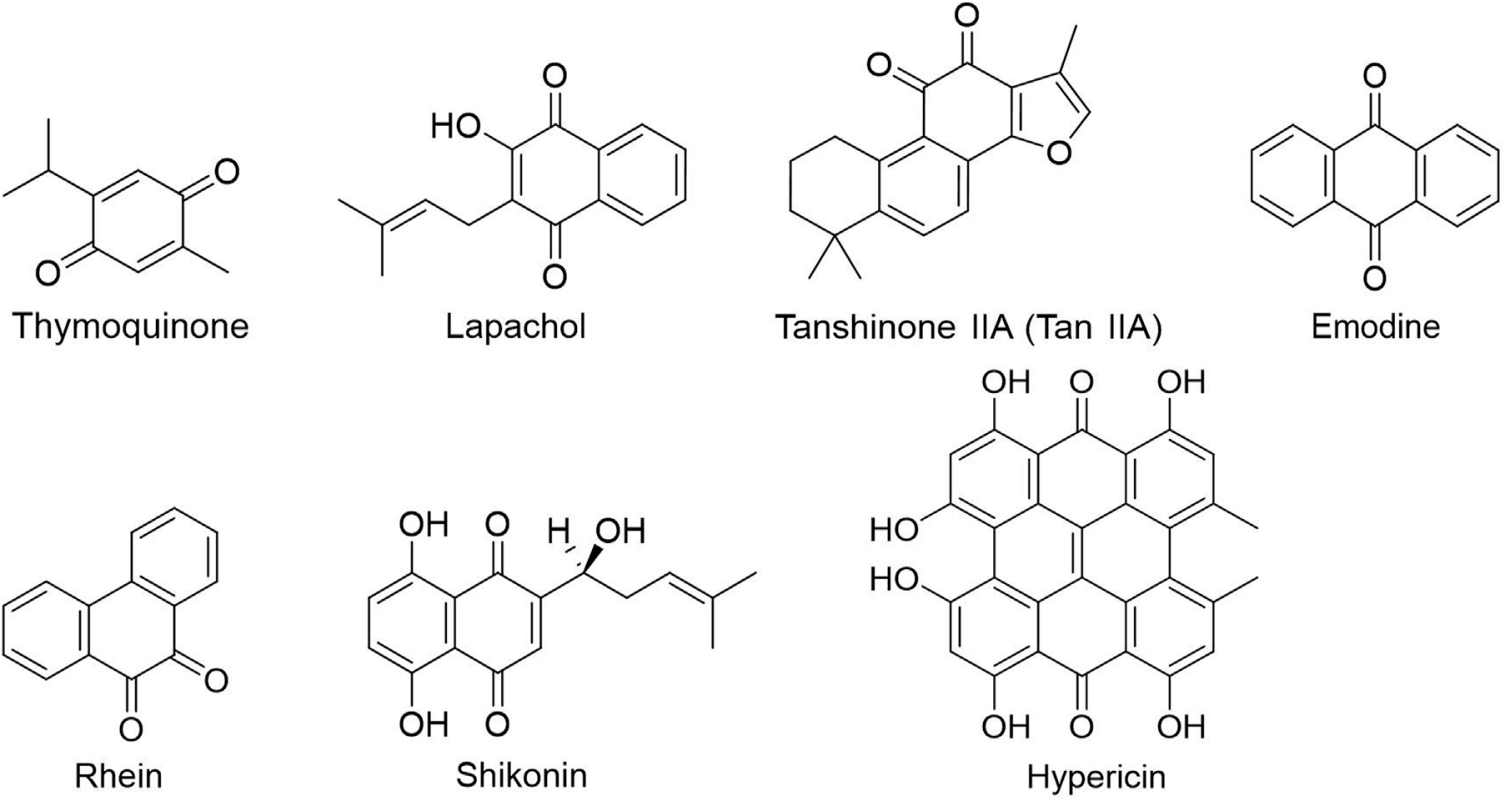
Quinones.

**FIGURE 6:**
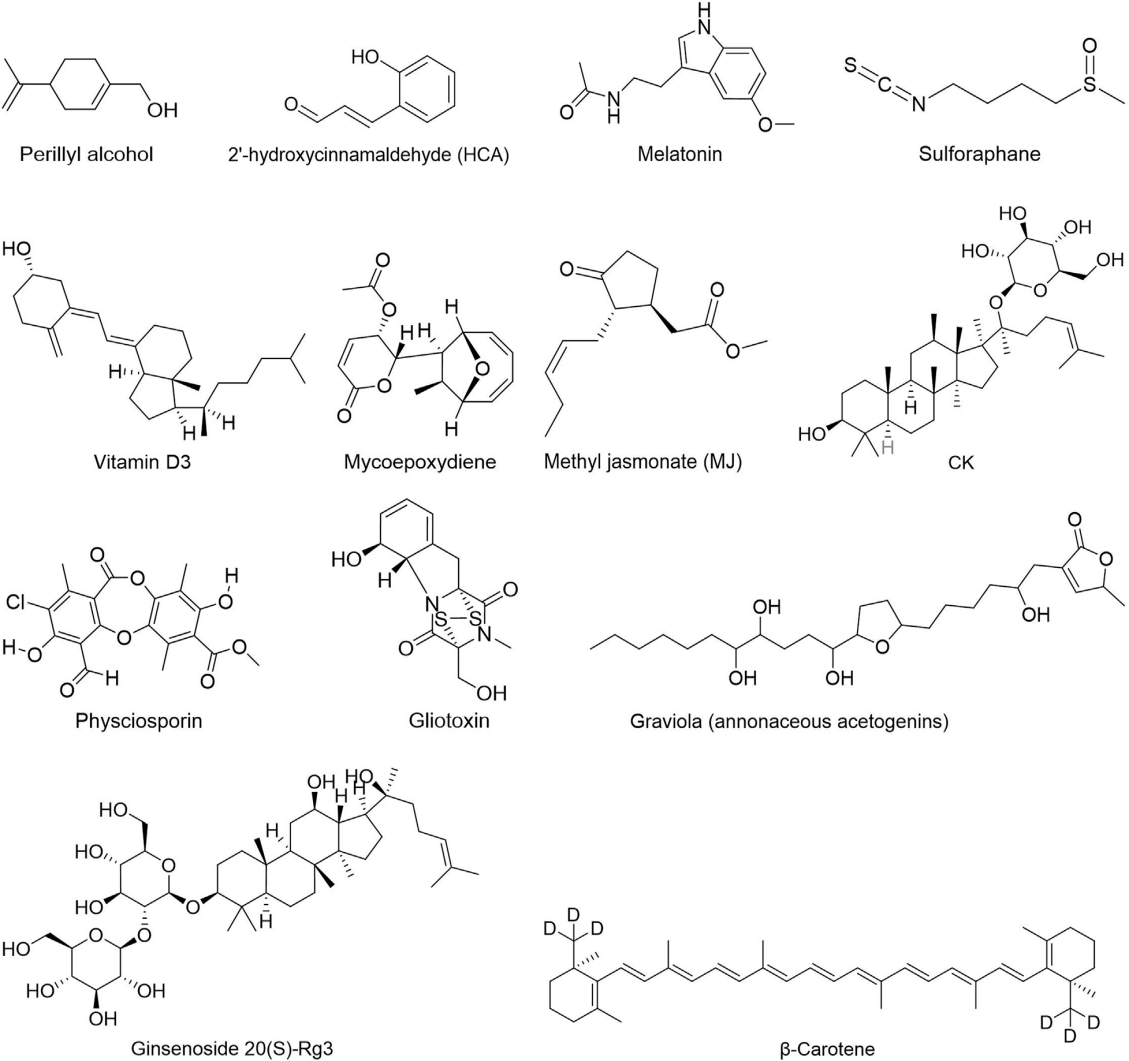
Others.

People have been using small-molecule drugs to treat tumors since the 1940s. A succession of medications, including alkylating agents, fluorouracil, methotrexate and cyclophosphamide, have been introduced to the market. While effective, most of these compounds also have severe adverse effect. Since the 1950s, drug researchers began to focus on natural small molecules for anti-cancer therapy and made great progress. Natural product is a compound or substance produced by living organisms in nature. According to their different structures, they can be divided into alkaloids (Figure 1), flavonoids (Figure 2), Non-flavonoid Phenolic Compounds (Figure 3), terpenoids (Figure 4), quinones (Figure 5) and others (Figure 6). Structures of natural compounds in each category were shown in Figures 1–6.

Phenols have been shown to have beneficial impacts on cancer prevention and treatment among other things. Phenols substances include flavonoids, tannins, phenolic acids, and anthocyanins, all of which include multiple phenolic hydroxyl groups in their chemical structure. Flavonoids are a kind of natural compounds with different phenolic structures. They usually exist in plants in the form of aglycones, glycosides and methylated derivatives. Usually the structure contains at least one hydroxyl aromatic ring, which has strong antioxidant activity. In addition, they have also been reported to play an important role in anti-bacterial, heart protection, anti-inflammatory and immune enhancement (Kumar and Pandey, 2013; Ahmed et al., 2016; Andreu et al., 2018; Tungmunnithum et al., 2018). Alkaloids are a class of nitrogen-containing organic compounds that exist mainly in plants in nature. They have antioxidant, anti-inflammatory, antimicrobial and anti-cancer effects both *in vivo* and *in vitro* (Kooshki et al., 2022). Alkaloids are a large group of complex natural compounds, which are widely distributed in nature and most found in the family of Ranunculaceae, Menispermaceae, and Papaveraceae. Alkaloids usually exist in the form of one or more nitrogen atom combinations, and have been proved to have anti-oxidative, anti-inflammatory, anti microbial and anti-cancer effects both *in vivo* and *in vitro* (Ahmad et al., 2017; Ahmad et al., 2020). Terpenoids are natural hydrocarbons that consists of five units of isoprene. Studies have shown that terpenoids play an important therapeutic role in anti-microbial, anti-hyperglycemia, anti-inflammatory and immune regulation (Shivapriya et al., 2011; Brahmkshatriya and Brahmkshatriya, 2013). Quinones are cyclic diketones with two double bonds and six carbon atoms, including benzoquinone, naphthoquinone, phenanthraquinone and anthraquinone. Resent research found quinones plays important role in anti-bacteria and anti-inflammation.

Chemical structures target glycolytic signaling pathway were shown in Figure 1.

## Natural products targeting glycolysis-related factors

### Inhibitors focus on hexokinase in glycolytic pathway

HK catalyzes the phosphorylation of the six hydroxyl group of hexose, which is the first step of glycolysis and plays an important role in regulating energy metabolism. In the past few years, studies identified that many of the natural compounds, as shown in (Table 1) were able to interfere with or inhibit the expression or activity of HK.

**TABLE 1:** Natural compounds target hexokinase (HK).

Chemical Class	Compound/extract name	Types of Study	Cancer model (s)	Effects on glycolysis and potential mechanisms of action	References
Alkaloids	Berberine	*In vitro*	Ovarian cancer	Inhibited the HK2 expression in SKOV3 and 3AO cells	Li et al. (2021)
	Dauricine (Dau)	*In vitro and in vivo*	Hepatocellular carcinoma	Inhibited the expression of HK2 and pyruvate kinase M2 (PKM2) in HepG2 and Huh-7 cells	Li et al. (2018a)
	Matrine	*In vitro and in vivo*	Colon cancer	Inhibited the mRNA and protein expression of HIF-1α and its downstream glucose metabolism-related enzymes GLUT1, HK2, and LDHA expressions in both HCT116 and SW620 cells	Hong et al. (2019)
	Sinomenine	*In vitro and in vivo*	Non-small cell lung cancer	Inhibited the expression of HK2 in HCC827, H1975, and H460 cells	Liu et al. (2020a)
	NK007	*In vitro*	Ovarian cancer	Suppressed the protein expression of HK2 in A2780 and A2780 (Taxol) cells	Li et al. (2019a)
Flavonoids	Chrysin	*In vitro and in vivo*	Hepatocellular carcinoma	Inhibited HK2 expression in HCC cell lines and HCC xenograft models	Xu et al. (2017)
	Quercetin	*In vitro and in vivo*	Hepatocellular carcinoma	Inhibited HK2 expression in both Bel-7402 and SMMC-7721 cells and SMMC-7721 xenograft model	Wu et al. (2019)
	Baicalein	*In vitro*	Gastric cancer	Suppressed the expression of HK2, LDHA and PDK under a hypoxic condition in AGS cells	Chen et al. (2015)
	Wogonin	*In vitro and in vivo*	Colon cancer, ovarian cancer, liver cancer	Decreased glucose uptake and PGM, HK2, GLUT1, PDHK1 and LDHA expressions in HCT116, A2780 and HepG2 cells	Zhao et al. (2018a)
		*In vitro and in vivo*	Colon Cancer	Inhibited the expression of HK2, PDK1, and LDHA in HCT116 cells and HCT116 xenograft model	Wang et al. (2014)
	Oroxylin A	*In vitro*	Breast cancer	Suppressed the transcription of HIF1α-targeted gene HK2 in MDA-MB-231 cells	Wei et al. (2015)
	Icaritin	*In vitro*	Glioblastoma	Inhibited the expression of HK2 in both U87 and T98G cells	Li et al. (2018b)
	Genistein	*In vitro and in vivo*	Hepatocellular carcinoma	Downregulates the expression of GLUT1 and HK2 to suppress aerobic glycolysis	Li et al. (2017a)
	Bavachinin	*In vitro and in vivo*	Cervical cancer and osteosarcoma	Decreased HIF1α expression and its hypoxia-inducible genes such as VEGF, GLUT1 and HK2 expressions in HOS and KB cells	Nepal et al. (2012)
	Xanthohumol	*In vitro and in vivo*	Colorectal cancer	inhibited glycolysis and the expression of HK2 in CRC cells and colorectal cancer athymic nude mouse model	Liu et al. (2019)
	Epigallocatechin-3-gallate (EGCG)	*In vitro and in vivo*	Breast cancer	Decreased the expression of HIF1α and GLUT1, and also Inhibited the activity of HK2, PFK, PK and LDH	Wei et al. (2018), Barbosa and Martel (2020)
	Deguelin	*In vitro and in vivo*	Non-small cell lung cancer	inhibited HK2 presence on mitochondrial outer membran and expression in H1650, H460 and HCC827 cells	Li et al. (2017b)
	Licochalcone A (LicA)	*In vitro and in vivo*	Gastric cancer	Inhibited HK2 expression in gastric cancer cell lines and gastric xenograft model	Wu et al. (2018a)
Non-flavonoid Phenolic Compounds	Resveratrol	*In vitro and in vivo*	Non-small cell lung cancer and hepatocellular carcinoma	Decreased glycolysis and HK2 expression	Dai et al. (2015), Li et al. (2016a), Brockmueller et al. (2021)
Quinones	Tanshinone IIA (Tan IIA)	*in vitro and in vivo*	oral squamous cell carcinoma	Suppressed the expression of HK2 in CAL27 and SCC15 cells and CAL27-derived xenograft tumors	Li et al. (2020a)
	Emodine and Rhein	*In vitro and in vivo*	Pancreatic cancer	Inhibited HIF-1α expression and the protein levesl of its downstream targets GLUT1, HK2 and PFK1 in MiaPaCa2 cells	Hu et al. (2017)
	Hypericin	*In vitro*	Glioma	Inhibited the mitochondria-bound hexokinase in a light- and dose-dependent manner	Miccoli et al. (1998)
Terpenoids	Dioscin	*In vitro and in vivo*	Colorectal cancers	Inhibited HK2 expression in HCT-116 and HT-29 cells	Wu et al. (2020)
	Jolkinolide B	*In vitro and in vivo*	Melanoma	Inhibited the mRNA expression of GLUT1, GLUT3, GLUT4, HK2 and LDHA in B16F10 cells	Gao et al. (2016)
	Limonin	*In vitro*	Hepatocellular carcinoma	Decreased glycolysis by inhibiting HK2 activity in HepG2 and Hep3B cells	Yao et al. (2018a)
	Oleanolic acid	*In vitro*	Gastric cancer	Decreased the expression and intracellular activities of HK2 and PFK1 in MKN-45 and SGC-7901 cells	Li et al. (2019b)
	Triptolide	*In vitro and in vivo*	Head and neck cancer	Suppressed the expression of HK2 in HK1, FaDu and C666-1 cells	Cai et al. (2021)
	β-escin	*In vitro and in vivo*	Ovarian Cancer	Inhibited the expression of HIF1α-targeted proteins, LDHA and HK2 in β-escin treatment omental tumors	Cheong et al. (2018), Kenny et al. (2021)
	Prosapogenin A (PSA)	*In vitro*	Breast cancer	Inhibited the expressions of GLUT1, HK and PFKL in MCF-7 cells	Wang et al. (2013a), Gao and Chen (2015)
Others	Methyl jasmonate (MJ)	*In vivo*	T Cell Lymphoma	Decreased the expression of HIF-1α, HK2, LDHA, PDK-1, and GLUT-1	Goel et al. (2021)
	Graviola (annonaceous acetogenins)	*in vitro and in vivo*	Pancreatic cancer	inhibited cellular metabolism and downregulatedthe mRNA expression of GLUT1, GLUT4, HK2 and LDHA in PC cells	Torres et al. (2012)
	Carpesium abrotanoides L. (CA)	*In vitro*	Breast cancer	Inhibited the expression of GLUT1, LDHA, and HK2	Chai et al. (2019)
	Mycoepoxydiene	*In vitro*	Cervical cancer	inhibited the protein levels of HK2, PFKM, ALDOA, ENO1 and LDHA, also supresses the LDHA and G6PD enzymatic activities in HeLa cells	Jin et al. (2017)
	Ganoderma sinense	*In vitro*	Pancreatic ductal adenocarcinoma	inhibited HK2 enzymatic activity	Bao et al. (2018)

**Alkaloids:** Berberine is a naturally occurring alkaloid derived from coptis, phellodendron, and three needles. In 2021, Li et al. (2021) demonstrated for the first time that berberine increased the inhibition of HK2 by miR-145 in OvCa SKOV3 and 3AO cells by boosting TET3-mediated demethylation of pre-miR-145, hence limiting Warburg of tumor cells. Dauricine, an alkaloid, can effectively decrease the expression of HK2 and PKM2 by up-regulating miR-199a, making hepatocellular carcinoma cells more sensitive to chemotherapy treatment (Li et al., 2018a). Matrine is a natural alkaloid derived from the roots of *Sophora flavescens* Ait, a traditional Chinese medicine. Matrine has been found in studies to drastically suppress the expression of HIF-1α and its downstream regulatory targets of glucose metabolism GLUT1, HK2 and LDHA in colon cancer HCT116 and SW620 cells, reversing the Warburg Effect (Hong et al., 2019). Sinomenine, taken from Sinomenium acutum, has been demonstrated *in vitro* and *in vivo* to have a pro-apoptotic impact and a substantial effect on NSCLC cells by inhibiting HK2-mediated glycolysis. NK007, a (±)‐tylophorine malate obtained from the Asclepiadaceae family, suppresses the proliferation of PTX-resistant ovarian cancer cells by reducing HK2-mediated glycolysis (Li et al., 2019a; Liu et al., 2020a).

**Flavonoids:** Chrysin is derived from blue passion flower, propolis and honey. Chrysin and quercetin can reduce the overexpression of HK2 in HCC cells and xenografts of HCC cells, limiting the growth of HCC cells dependent on aerobic glycolysis (Xu et al., 2017; Wu et al., 2019). Baicalin, wogonin, and oroxylin A are scutellaria baicalensis-extracted flavonoids. Through research on stomach cancer, it was discovered that baicalin inhibits the expression of three important glycolysis enzymes, HK2, LDHA, and PDK, reducing the rate of glycolysis and reversing the hypoxia-induced sensitivity of AGS cells to pentafluorouracil (Chen et al., 2015). Wogonin inhibits gene expression of glycolytic associated factors like HK2 and LDHA in ovarian and liver cancer cells, hence limiting glycolysis and cell proliferation (Zhao et al., 2018a). Moreover, *in vitro* and *in vivo* investigations on colon cancer showed that wogonin inhibited HIF-1α, HK2, PDK1, and LDHA expression (Wang et al., 2014). Oroxylin A suppresses the glycolysis-dependent proliferation of breast cancer MDA-MB-231 cells by activating SRT3, and decreasing HIF-1α-targeted HK2 gene transcription (Wei et al., 2015). Icariin is a pentene flavonoid derivative. Li et al. (2018b) discovered that icariin inhibits the expression of HK2 in glioblastoma U87 and T98G cells, and reduces glucose consumption and lactate production. Genistein, an isoflavone molecule obtained from soybeans, and Bavachinin, a flavonate isolated from *Proralea Corylifolia L*. seeds, were reported to inhibit GLUT1 and HK2 in HCC, HOS and KB cells, respectively, *via* lowering the expression and activity of HIF-1α, reducing glycolysis and promoting apoptosis in hepatocellular carcinoma cells, as well as inhibiting angiogenesis in cervical cancer and osteosarcoma (Nepal et al., 2012; Li et al., 2017a). According to Liu et al. (2019), Xanthohumol can enhance cytochrome c release and activate the intrinsic apoptotic pathway in human colorectal cancer cells and xenografts by suppressing HK2 overexpression (Liu et al., 2019). Wei et al. (2018) reported that epigallocatechin-3-gallate (EGCG), a green tea polyphenol, could inhibit the activity of HK2, PFK and LDH in the glycolytic signaling pathway, resulting in cell apoptosis and autophagy in breast cancer cells both *in vitro* and *in vivo* (Barbosa and Martel, 2020). Deguelin, a natural compound derived from the African plant *Mundulea sericea,* could inhibit glucose metabolism by reducing Akt-mediated HK2 over-expression in NSCLC cell line while also inhibit HK2 localization in mitochondria outer membrane, resulting in decreased glycolysis and induction of apoptosis (Li et al., 2017b). Licochalcone A, a chalcone derived from liquorice, inhibit the expression of HK2 *via* the Akt signaling pathway and are effective in human non-small cell lung cancer (NSCLC) and gastric cancer (Wu et al., 2018a).

**Non-flavonoid Phenolic Compounds:** Resveratrol, a dietary polyphenol derived from grapes, peanuts, mulberries, and other fruits and vegetables could inhibit the expression of HK2 *via* the Akt signaling pathway and are effective in NSCLC and gastric cancer. Another study found that resveratrol-induced mitochondrial apoptosis in HCC cells was linked to a reduction in HK2 expression (Dai et al., 2015; Li et al., 2016a; Brockmueller et al., 2021).

**Quinones:** Quinone chemicals are a type of chemical components that have a quinoid structure and are classified as benzoquinone, naphthoquinone, phenanthrenequinone, and anthraquinone. Tanshinone IIA, the main component of Danshen (*Salvia miltiorrhiza Bunge*), was confirmed by Li et al. (2020a) to promote the E3 ligase FBW7-mediated ubiquitination and degradation of c-Myc by inhibiting the Akt-c-Myc signaling pathway, ultimately reducing the expression of HK2 has become one of the potential anti-oral squamous cell carcinoma drugs. Emodin and rhein are anthraquinone components derived from *Rheum palmatum* that can inhibit HIF-1α and diminish the downstream glucose regulating molecules, GLUT1, as well as reduce the expression of HK2 and PFK1 and prevent the Warburg Effect of human pancreatic cancer cells (Hu et al., 2017). Natural photosensitizer hypericin inhibited mitochondria-bound hexokinase in a light and dose dependent manner, thus inhibiting energy consumption in human glioma cells (Miccoli et al., 1998).

**Terpenoids:** Dioscin is a steroid saponin isolated from the rhizomes of *Dioscoreae* and *Paridis*. Wu et al. (2020) showed that dioscin inhibits HCT-116 and HT-29 cells by increasing FBW-7-mediated c-myc ubiquitination and the activity of HK2, which inhibits glycolysis and promotes apoptosis. B16F10 cells may manufacture Jolkinolide B (JB), a bioactive diterpenoid isolated from the root of *Euphorbia fischeriana Steud*, by down-regulating the mRNA expression of glycolysis-related genes HK2 and LDHA and glucose transporters GLUT1, GLUT3, and GLUT4, promoting melanoma cell apoptosis (Gao et al., 2016). Limonin is primarily found in citrus fruits such as lemons, oranges, pummelo, grapefruits, bergamots and mandarins. In hepatocellular carcinoma, limonin promotes a translocation of HK2 from mitochondria to the cytoplasm by inhibiting Akt-mediated phosphorylation of HK2, thereby reducing HK2 activity, further activating BAX, and causing the release of the apoptotic factor cytochrome c (Yao et al., 2018a). Oleanolic acid, a triterpenoid component found in vegetable oils and the leaves or roots of Oleaceae family plants, can suppress cancer cell growth by reducing the expression and activity of HK2 and PFK1 in human gastric carcinoma cells (Li et al., 2019b). Triptolide (TPL), a natural diterpenoid epoxide derived from a traditional Chinese herb, reduced the expression of c-myc and mitochondrial HK2 in head and neck cancer cells and activated the BAD/BAX-caspase 3-GSDME cascade, triggering GSDME-mediated pyroptosis (Cai et al., 2021). β-Escin is a natural pentacyclic triterpenoid saponin derived from the seed of *Aesculus hippocastanum L*. It has been found in studies to limit the lowering of HIF-1α-targeted proteins, LDHA, CD31, and HK2 in ovarian cancer (OvCa) mouse omentum, therefore inhibiting OvCa invasion (Cheong et al., 2018; Kenny et al., 2021). Prosapogenin A (PSA), a natural product of Veratrum, increases MCF-7 cell death by inhibiting STAT3 and glucose metabolism-related genes GLUT1, HK and PFKL (Wang et al., 2013a; Gao and Chen, 2015).

**Others:** Several natural chemicals play a vital role in tumor glycolysis. A study demonstrated that Methyl jasmonate (MJ), a natural oxylipin, inhibits glucose metabolism regulatory molecules, namely HIF-1α, HK2, LDHA, PDK1 and GLUT1, for the first time *in vitro* in Dalton’s lymphoma, providing a novel anticancer therapy for the treatment of hematological malignancies (Goel et al., 2021). Graviola, which is extracted from *Annona Muricata*, could inhibit glucose uptake in pancreatic cancer cells by suppressing the expression of HIF-1α, NF-κB, GLUT1/GLUT4, HK2 and LDHA according to a recent study, thereby decrease ATP generation (Torres et al., 2012). Carpesium abrotanoides L (CA) and mycoepoxydiene (MED) were reported to have anti-breast cancer role and could inhibited cervical cancer progression by suppressing the expression of glycolysis-related genes GLUT1 and HK2 (Jin et al., 2017; Chai et al., 2019). A novel steroid from *Ganoderma sinense*, (22*E*,24*R*)-6β-methoxyergosta-7,9(11), 22-triene3β,5α-diol (2), inhibited HK2 activity in human pancreatic ductal adenocarcinoma (PDAC) SW1990 cells (Bao et al., 2018).

### Inhibitors focus on phosphofructokinase 1 in glycolytic pathway

PFK-1 is the second rate-limiting enzyme in glycolysis and its activity is mainly regulated by PFKFB3, which catalyzes the formation of fructose 2,6-diphosphate from fructose 6-phosphate rather than directly participate in the catalytic process of glycolysis. Natural compounds target phosphofructokinase 1 (PFK1) or PFKFB3 were shown in (Table 2).

**TABLE 2:** Natural compounds target phosphofructokinase 1 (PFK1).

Chemical Class	Compound/extract name	Types of Study	Cancer model (s)	Effects on glycolysis and potential mechanisms of action	References
Alkaloids	Worenine	*in vitro*	Colorectal cancer	Downregulated PFK-L, HK2 and PKM2 gene expression in HCT116 and SW620 cells	Ji et al. (2021)
	Berberine	*in vitro*	Breast cancer	Inhibited the protein levels of PFKP in MCF-7 cells	Tan et al. (2015)
Flavonoids	epigallocatechin-3-gallate (EGCG)	*in vitro and in vivo*	Breast cancer	Directly suppressed PFK activity by converting the tetrameric structure of PFK to its inactive form in HCC-LM3, SMMC-7721, Hep3B, HepG2, and Huh-7 cell lines	Li et al. (2016b)
		*in vitro and in vivo*	Breast cancer	Suppressed mRNA levels of HK, PFK and LDH, and PK activity in 4T1 cells	Wei et al. (2018)
Non-flavonoid Phenolic Compounds	Curcumin	*in vitro*	Esophageal cancer	Induced significant downregulation of GLUT4, HK2, PFKFP3, and PKM2 protein expression in Ec109 cells	Zhang et al. (2015)
	Resveratrol	*in vitro*	Breast cancer	Suppressed PFK activity, promoting the dissociation of PFK from a highly active tetramer to a less active dimer in MCF-7 cells	Gomez et al. (2013)
		*in vitro*	Human GC-like DLBCL cancer	Suppressed aerobic glycolysis by reducing mRNA levels of HK2 and PFK1 in OCI-Ly1 (LY1) and OCI-Ly18 (LY18) cells	Faber et al. (2006)
Quinones	Rhein and Emodine	*In vitro and in vivo*	Human pancreatic cancer Line (MIAPaCa-2)	The expression of Glut1, HK2, and PFK1 is suppressed and the level of HIF-1α is lowered in MiaPaCa2 cells	Hu et al. (2017)
	Shikonin	*in vitro*	Lung cancer	Downregulated the expression of PFKFB2 in A549 and H446 cells	Sha et al. (2021)
Terpenoids	Oleanolic acid	*in vitro*	Gastric cancer cells	OA also decreased the expression and intracellular activities of HK2, HIF-1α and PFK1 in MKN-45 and SGC-7901 cells	Li et al. (2019b)

**Alkaloids:** As shown in HCT116 and SW620 cells, Worenine inhibited glycolysis, cellular energy generation, and macromolecule manufacturing by downregulating PFK-L, HK2 and PKM2 gene expression (Ji et al., 2021). Berberine suppressed glycolysis in human breast cancer MCF-7 cells by modulating ATP content and pH value (Tan et al., 2015).

**Flavonoids:** EGCG strongly suppressed mRNA levels of HK, PFK and LDH, and PK activity to a lesser extent in breast cancer cells (4T1). Furthermore, EGCG inhibited glucose metabolism and had an anticancer effect *via* suppressing the expression of HIF-1α and GLUT1 (Wei et al., 2018). Another study showed that EGCG directly suppressed PFK activity by converting the tetrameric structure of PFK to its inactive form, resulting in the inhibition of glycolysis and the eventual death of tumor cells (HCC-LM3, SMMC-7721, Hep3B, HepG2, and Huh-7 cell lines). As an added bonus, EGCG boosted sorafenib’s antitumor effect, meaning it could be utilized in combination therapy to overcome sorafenib’s resistance issue (Li et al., 2016b).

**Non-flavonoid Phenolic Compounds:** Curcumin inhibited the growth of human esophageal cancer Ec109 cells *via* activating the AMPK signaling pathway, which in turn reduced the mRNA and protein expressions of GLUT4, HK2, PFKFB3 and PKM2, hence reduced the Warburg Effect of Ec109 cells (Zhang et al., 2015). The enzymatic activity of PFK was directly inhibited by resveratrol in MCF-7 cells by promoting the dissociation of PFK from a highly active tetramer to a less active dimer, resulting in decreased glucose consumption and ATP content, and ultimately tumor cell death. The decline in vitality was statistically significant (Gomez et al., 2013). Resveratrol also suppressed aerobic glycolysis by reducing mRNA levels of HK2 and PFK1 in the human GC-like DLBCL cell lines OCI-Ly1 (LY1) and OCI-Ly18 (LY18) (Faber et al., 2006).

**Quinones:** The expression of GLUT1, HK2, and PFK1 is suppressed and the level of HIF-1α is lowered by emodin and rhein in human pancreatic cancer cells (MiaPaCa2) (Hu et al., 2017). Shikonin directly downregulates the expression of PFKFB2 in human lung cancer cells (A549 and H446), inhibiting the Warburg Effect and exerting anticancer action in lung cancer cells (Sha et al., 2021).

**Terpenoids:** Oleanolic acid (OA) inhibited tumor growth in human gastric cancer cells (MKN-45 and SGC-7901) by decreasing HK2 and PFK1 expression and intracellular activity, as well as decreasing HIF-1α expression and nuclear abundance, hence reduced glycolysis and induced cell apoptosis (Li et al., 2019b).

### Inhibitors focus on pyruvate kinase in glycolytic pathway

PK is the third rate-limiting enzyme in glycolysis. It catalyzes the last process in the conversion of glucose to pyruvate and simultaneously transfers the phosphate group from phosphoenolpyruvate to ADP to generate pyruvate. Natural compounds target PK were shown in (Table 3).

**TABLE 3:** Natural compounds target pyruvate kinase (PK).

Chemical Class	Compound/extract name	Types of Study	Cancer model (s)	Effects on glycolysis and potential mechanisms of action	References
Alkaloids	Berberine	*in vitro*	Colon cancer and cervical cancer	Inhibited the enzyme activity of PKM2 in HCT116 and HeLa cells	Li et al. (2017c)
	Dauricine	*in vitro and in vivo*	Hepatocellular carcinoma	Downregulated the expression of HK2 and PKM2 in HCC cells, and sensitized HCC cells to sorafenib in a xenograft mouse model Dau sensitized HCC cells to sorafenib.	Li et al. (2018a)
	N-methylhemeanthidine	*in vitro and in vivo*	Pancreatic cancer	Inhibited the protein levels of GLUT1, PGK1, LDHA, and PKM2 in AsPC-1, BxPC-3 and Mia PaCa-2 cells	Guo et al. (2014)
	Oxymatrine	*in vitro and in vivo*	Colorectal cancer	The PKM2 kinase activity and expression were inhibited by oxymatrine in CRC cells, inhibited PKM2 and GLUT1 expression in a xenograft mouse model	Li et al. (2020b)
Flavonoids	Apigenin (AP)	*In vitro*	Colon cancer	Inhibited PKM2 protein level and activity in HCT116, HT29 and DLD1 cells	Shan et al. (2017)
	Catechin derivatives	*In vitro*	Cervical cancer	Inhibited PKM2 enzyme activity in HeLa cells	Adem et al. (2016)
	Isovitexin (IVT)	*in vitro and in vivo*	Lung cancer	Downregulated the protein expressions of PKM2 in A549 and H1975 cells	Chen et al. (2021a)
	Kaempferol	*In vitro*	Colon cancer	Promoted the expression of miR-339-5p, reduced the expression of PKM2 but induced that of PKM1 in HCT-116 and DLD1 cells	Wu et al. (2021)
	Naringin	*In vitro*	Malignant melanoma	Inhibited the expression of PKM2, LDHA, and HIF-1α in A375 cells	Guo et al. (2016)
	Oroxylin A	*in vitro*	Hepatocellular carcinoma	Inhibited Hif1α, HK2, LDHA, PDK, PKM2 mRNA and protein level in HepG2 cells	Wei et al. (2017)
	Morusin	*In vitro*	Hepatocellular carcinoma	Attenuated the expression of AKT, mTOR, c-Myc, HK2, PKM2, and LDH in Hep3B and Huh7 cells	Cho et al. (2021)
	Proanthocyanidin B2	*in vitro and in vivo*	Hepatocellular carcinoma (HCC)	Inhibited the expression and nuclear translocation of PKM2, therefore disrupting the interaction between PKM2/HSP90/HIF-1α in five kinds of HCC cell lines	Feng et al. (2019)
	Scutellarin	*In vitro*	Cervical cancer	Inhibits its cytosolic activity and promote the nuclear translocation of PKM2 in Hela cells	You et al. (2017)
	Quercetin	*in vitro and in vivo*	Breast cancer	Decreased the level of glycolysis-related proteins PKM2, GLUT1 and LDHA in MCF-7 and MDA-MB-231 cells, reduced the level of PKM2 in a xenograft mouse model	Jia et al. (2018)
Non-flavonoid Phenolic Compounds	Curcumin	*In vitro*	Lung cancer, breast cancer, cervical cancer and prostate cancer and human embryonic kidney cancer	Downregulated the expression of PKM2, via inhibition of mTOR-HIF1α axis in HEK293, H1299, MCF-7, HeLa and PC3 cell lines	Siddiqui et al. (2018)
		*In vitro*	Head and neck squamous cell carcinomas	Greatly suppressed the expression of PKM2 in FaDu, Detroit 562, and Cal27 cells	Mojzes et al. (2020)
	Resveratrol	*in vitro*	Liver cancer, breast cancer, cervical cancer	Downregulated PKM2 expression by inhibited mTOR signaling and suppressed cancer metabolism in HepG2, MCF7, Hela cells	Iqbal and Bamezai, (2012)
		*in vitro*	Colon cancer, cervical cancer, and breast cancer	Decreased the expression of PKM2 in DLD1, HeLa, and MCF-7 cells	Wu et al. (2016)
	Tannic acid	*in vitro*	Colon cancer	Inhibited the pyruvate kinase activity of PKM2, rather than protein kinase activity and PKM2 expression, and had no effect on PKM1 activity in DLD1, HCT-116 cells	Yang et al. (2018)
Quinones	Lapachol	*in vitro*	Melanoma	Inhibited PKM2 activity of purified enzyme in MEL526, MEL697, MEL103 and A375 cell lines	Shankar Babu et al. (2018)
	Shikonin	*in vitro*	Skin cancer	Inhibited oncogenic activation, PKM2 activation, and mitochondrial dysfunction in Murine skin epidermal JB6 Cl-41 (Pþ) cells	Li et al. (2014)
		*in vitro*	Skin tumor	Inhibited the activity of PKM2 in Murine skin epidermal JB6 Cl-41 (P+) cells	Li et al. (2015a)
		*In vitro/ in vivo*	Hepatocellular Carcinoma	Inhibited the level of PKM2 in LM3, SMMC-7721, Huh-7, and HepG2 cells. SK combined with sorafenib markedly inhibits tumor growth in a xenograft mouse model	Liu et al. (2020b)
		*In vitro*	Hepatocellular carcinoma	Inhibited the expression of PKM2 in HCC cells	Chen et al. (2011)
		*in vitro and in vivo*	Non-small cell lung cancer	Induced cell cycle arrest and apoptosis by inhibiting PKM2/STAT3/cyclinD1 signal pathway in vitro and in vivo.	Tang et al. (2018)
		*in vitro and in vivo*	Lewis lung carcinoma (LLC) and melanoma	Reduced the level of tumor cell PKM2 phosphorylation though in B16 and MKN-45 cells	Zhao et al. (2018b)
		*in vitro and in vivo*	Non-small cell lung cancer (NSCLC)	Downregulated the expression of PKM2 and Glut1 in A549 and PC9 cells	Dai et al. (2022)
	Tanshinone ⅡA	*in vitro*	Esophagus cancer cell	Inhibited human esophagus cancer cell growth through miR-122-mediated PKM2 down-regulation pathway in Ec109 cells	Zhang et al. (2016)
Terpenoids	Cantharidin	*in vitro and in vivo*	Breast cancer	Prevented the translocation of PKM2 into the nucleus in MDA-MB-231 and MCF-7 cells	Pan et al. (2019)
	Dihydroartemisinin	*in vitro*	Esophageal cancer	Downregulated the expression of PKM2 in Eca109 and Ec9706 cells	Li et al. (2019c)
	Cynaropicrin	*In vitro*	Lung cancer	Inhibited the purified PKM2 activity and decreased the cellular PKM2 expression in A549 cells	Ding et al. (2021)
	Micheliolide	*in vitro and in vivo*	Leukemia	Targeted the PKM2 residue C424, promoted tetramer formation, and inhibited PKM2 nuclear translocation in HL-60 cells	Li et al. (2018c)
	Oleanolic acid	*in vitro*	Prostate cancer and breast cancer	Induced a switch from PKM2 to PKM1, and abrogated Warburg effect in PC3 and MCF7 cells	Liu et al. (2014)
		*in vitro*	Breast cancer	Decreased the protein levels of HK, PKM2, and LDHA in MDA-MB-231 cells	Amara et al. (2016)
	Ursolic acid	*in vitro*	Breast cancer	Decreased the levels of HK2 and PKM2 in SK-BR-3 and MCF-7 cells	Lewinska et al. (2017)
Others	2'-hydroxycinnamaldehyde (HCA)	*in vitro and in vivo*	Prostate cancer	Induced PKM2 to exist in a tetrameric form with strong PK activity and inhibited PKM2-mediated STAT3 phosphorylation in DU145 cells	Yoon et al. (2018)
	CK	*in vitro*	Hepatocellular carcinoma	Suppressed AKT/mTOR/c-Myc, HK2 and PKM2 in HepG2 and Huh7 cells	Shin et al. (2021)
	Ginsenoside 20(S)-Rg3	*in vitro and in vivo*	Ovarian cancer	Inhibited the expression of HK2 and PKM2 in SKOV3 and 3AO cells	Li et al. (2015b)
	Gliotoxin	*In vitro*	Glioma cancer	Bounded to PKM2, inhibited its tyrosine kinase and glycolytic activity in U87 cells.	Tang et al. (2020)
	Physciosporin	*in vitro and in vivo*	Breast cancer	Altered the levels of PGC-1α, GLUT1, HK2 and PKM2, and downregulated β-catenin, c-Myc, HIF-1α, and NF-κB in MCF-7 and MDA-MB-231 cells	Tas et al. (2021)

**Alkaloids:** Berberine, one of the alkaloids, suppressed tumor cell proliferation in HCT-116 and HeLa cells by blocking PKM2 enzymatic activity (Li et al., 2017c). Dauricine (Dau), another alkaloid, was found to efficiently decrease HK2 and PKM2 gene expression in hepatocellular carcinoma HCC cells by increasing the expression of miR-199a, hence increasing the chemosensitivity of HCC cells to some chemotherapy medicines like cisplatin and sorafenib (Li et al., 2018a). Protein levels of GLUT1, PGK1, LDHA, and PKM2 were all decreased by N-methylhemeanthidine chloride (NMHC) in pancreatic cancer cell lines (AsPC-1, BxPC-3, and Mia PaCa-2) (Guo et al., 2014), resulting in a decrease in glucose metabolism. Oxymatrine suppressed the metastasis of HT-29 and HCT-116 cells by reducing PKM2 expression and GLUT1 activity (Li et al., 2020b).

**Flavonoids**: Apigenin served as a new allosteric inhibitor that directly bound to PKM2 in HCT116 cells, greatly reducing PKM2 expression and activity. In addition, by inhibiting the catenin/c-Myc/PTBP1 signaling pathway, AP may assure low expression of PKM2/PKM1 in HCT116 cells (Shan et al., 2017). In a separate study, researchers examined the *in vitro* effects of 22 flavonoid derivatives on PKM2 enzyme activity. Results indicated that catechin derivatives may be employed as lead molecules in the design of PKM2 enzyme activators and inhibitors (Adem et al., 2016). Isovitexin (Apigenin-6-C-glucoside, IVT) inhibited the proliferation and glucose metabolism of A549 and H1975 cells by down-regulating the expression of PKM2 and its downstream factors (such as STAT3, Bcl-2, and Bcl-xl). Furthermore, IVT and cisplatin enhanced the inhibitory effect of tumor cells in a synergistic manner (Chen et al., 2021a). Kaempferol inhibited hnRNP family hnRNPA1 and PTBP1-induced altered splicing of PKM gene in HCT116 and DLD1 cells, resulting in the reduction of PKM2 and elevation of its isoenzyme PKM1 (Wu et al., 2021). Naringin could suppress gene expression of c-Src, PKM2, LDHA, and HIF-1α in A375melanoma cells through inhibiting the c-Src/Akt signaling pathway (Guo et al., 2016). Oroxylin A might block the production of polypyrimidine tract-binding protein and increase the ratio of PKM1/PKM2 in HepG2 and SMMC-7721 hepatoma cells (Wei et al., 2017). Morusin significantly induced AMPK/ACC phosphorylation and suppressed p-AKT, p-mTOR, c-Myc, HK2, PKM2, and LDH gene expression in hepatocellular carcinoids Huh7 and Hep3B cells, consequently exerting anticancer effects *via* AMPK-mediated G1 arrest (Cho et al., 2021). In five primary HCC cell lines, PB2 disrupted the PKM2/HSP90/HIF-1α connection by reducing PKM2 production and nuclear translocation, thereby inducing cell death *via* HIF-1α-mediated transcriptional repression (Feng et al., 2019). Scutellarin decreased the level of aerobic glycolysis in cervical cancer cells (Hela) by directly targeting PKM2 and blocking its cell membrane function. In addition, it may potentially enhance the nuclear translocation of PKM2 by stimulating the MEK/ERK/PIN1 signaling pathway, which regulates cell cycle and apoptosis proteins (You et al., 2017). In MCF-7 and MDA-MB-231 cells, quercetin inhibited the expression of cell migration marker proteins such as matrix metalloproteinase (MMP)-2, MMP-9 and vascular endothelial growth factor (VEGF), as well as PKM2, GLUT1 and LDHA, and successfully blocked cellular glycolysis by inhibiting glucose uptake and lactate production (Jia et al., 2018).

**Non-flavonoid Phenolic Compounds:** Curcumin decreased glucose uptake and lactate formation in cancer cell lines (H1299, MCF-7, HeLa, and PC3) *via* blocking the mTOR-HIF-1α interaction system and down-regulating PKM2 expression (Siddiqui et al., 2018). In cell lines originating from head and neck squamous cell carcinomas (FaDu, Detroit 562, and Cal27), tumor cells were particularly sensitive to ethanol-desolved curcumin, which greatly suppressed the expression of PKM2 (Mojzes et al., 2020). Resveratrol inhibited the mTOR signaling pathway in several cancer cells (HeLa, HepG2, and MCF-7) resulting in the downregulation of the mRNA and protein levels of PKM2 (Iqbal and Bamezai, 2012). Resveratrol also enhanced the expression of microRNA-326 (miR-326) and decreased the expression of PKM2 in DLD1, HeLa, and MCF-7 cells, triggering ER stress and mitochondrial functional impairment (Wu et al., 2016). Tannins acid (TA) suppressed the proliferation of human rectal cancer cells (DLD1 and HCT-116) *via* reducing PKM2 and PK activity. The underlying mechanism could be that TA directly binds to the K433 residue, which is a lysine residue and a pharmacologically acceptable site for selectively inhibiting PKM2. This disrupts the conformation of PKM2 tetramers and stops CRC cells from growing (Yang et al., 2018).

**Quinones:** Lapachol inhibited glycolysis by reducing PKM2 activity in melanoma cells (MEL526, MEL697, MEL103 and A375), resulting in lower ATP levels, suppression of cell growth, and promotion of apoptosis (Shankar Babu et al., 2018). Shikonin suppressed tumor promoter 12-O-tetradecanoylphorbol 13-acetate (TPA) caused tumor cell transformation and PKM2 activation in early stages of carcinogenesis in Skin Epidermal JB6 Cells, showing the chemopreventive potential of PKM2-for human skin cancer (Li et al., 2014). Shikonin has been shown to reduce skin carcinogenesis by blocking the transcription factor ATF2 pathway according to relative study (Li et al., 2015a). Shikonin suppressed tumor cell proliferation in HCC, LM3, SMMC-7721, Huh-7 and HepG2 cells *via* reducing the expression of PKM2 and cyclinD1, resulting in a decrease in PKM2 expression and the binding of PKM2 to Bcl-2, inducing apoptosis in HCC cells (Liu et al., 2020b). Shikonin inhibited glycolysis by inhibiting PKM2 in drug-sensitive and resistant cancer cells (MCF-7, MCF-7/Adr, MCF-7/Bcl-xL, MCF-7/Bcl-2 and A549), but had no effect on the expression of PKM1 and PKL (Chen et al., 2011). Shikonin promoted cell cycle arrest and death by blocking the PKM2/STAT3/cyclinD1 signaling pathway in human NSCLC cell lines A549, H1299, H1975 and HCC827, thereby increasing the anticancer impact of gefitinib both *in vitro* and *in vivo* (Tang et al., 2018). In melanoma B16 cells and gastric cancer MKN-45 cells, shikonin promoted tumor cell apoptosis by inhibiting the phosphorylation of PKM2 and preventing the conformational transition of PKM2 tetramer to dimer, but did not reduce total levels of PKM2 (Zhao et al., 2018b). Shikonin turned down the expression of PKM2 and its transcriptionally regulated downstream gene GLUT1 in NSCLC, A549 and PC9 cells, which inhibited tumor cells from grwoing, spreading, invading and migrating, and caused cell death. Shikonin could also make cisplatin work better on tumor cells by reducing the amount of exosomal PKM2 that is released from cells (Dai et al., 2022). Tanshinone IIA targeted PKM2 directly in human esophageal cancer Ec109 cells. This resulted in miR-122 being more active, which in turn prevented the proliferation of tumor cells (Zhang et al., 2016).

**Terpenoids:** In MDA-MB-231 and MCF-7 cells, cantharidin suppressed the progression of breast cancer by preventing the translocation of PKM2 into the nucleus, hence decreasing the activity of PK and cell migration and invasion (Pan et al., 2019). In human esophageal squamous carcinoma Eca109 and Ec9706 cell lines, dihydroartemisinin (DHA) may inhibit the glycolysis of esophageal cancer by downregulating PKM2 expression, hence decreasing tumor cell proliferation (Li et al., 2019c). Cynaropicrin (CYN) suppressed PKM2 expression and activity in lung cancer A549 cells, resulting in the up-regulation of p53 and down-regulation of PARP, followed by cell cycle arrest. In addition, CYN blocked the interaction between PKM2 and Nrf2, resulting in a reduction of cellular antioxidant capacity, oxidative stress, and mitochondrial damage (Ding et al., 2021). Micheliolide (MCL) specifically targeted the PKM2 residue C424, promoted tetramer formation, inhibited lysine 433 (K433) acetylation and inhibited PKM2 nuclear translocation in HL-60 cells, consequently reducing the proliferation and carcinogenesis of leukemia cells (Li et al., 2018c). OA inhibited aerobic glycolysis in human prostate carcinoma PC-3 cells and MCF-7 cells by inhibiting PKM2 expression. In addition, OA interfered with the mTOR/c-Myc/PKM2 pathway, switching metabolic mode from aerobic glycolysis to oxidative phosphorylation (Liu et al., 2014). In MDA-MB-231 cells, OA was able to efficiently suppress the expression and activity of high-salt-induced glycolytic rate-limiting enzymes HK, PK and LDH, suggesting the substance has a protective impact in breast cancer (Amara et al., 2016). By inhibiting the activity of AKT, ursolic acid (UA) decreased the levels of HK2 and PKM2 in human breast cancer SK-BR-3 and MCF-7 cells. This resulted in ATP and lactate deficiency in breast cancer cells, thereby inhibiting tumor cell proliferation and promoting cell apoptosis (Lewinska et al., 2017).

**Others:** By directly binding to PKM2 in prostate cancer cells (DU145), 2′-hydroxycinnamaldehyde (HCA) induced PKM2 to exist in a tetrameric form with strong PK activity and inhibited PKM2-mediated STAT3 phosphorylation, limiting cell proliferation *in vitro* and tumor growth *in vivo* (Yoon et al., 2018). Compound K (CK), a ginsenosides metabolite, reportedly possessed anticancer and antiangiogenic properties. CK triggered apoptosis in human hepatocellular carcinoma HCCs, HepG2 and Huh7 cells *via* the AKT/mTOR/c-Myc/HK2/PKM2 pathway (Shin et al., 2021). Ginsenoside 20(S)-Rg3 was another ginsenoside extract with similar biological activity as CK. By inhibiting HK2 and PKM2 in human ovarian cancer SKOV3 and 3AO cells, Ginsenoside 20(S)-Rg3 decreased tumor cell glycolysis (Li et al., 2015b). Gliotoxin selectively bind to PKM2 and inhibit glycolysis in human glioma U87 cells, resulting in decreased glucose intake and lactate generation in tumor cells (Tang et al., 2020). In MCF-7 and MDA-MB-231 cells, physciosporin (PHY) at high doses affected Bcl-2 and activated apoptosis. Low concentrations of PHY downregulated the amounts of rate-limiting enzymes PGC-1 in the respiratory chain, GLUT1, HK2, and PKM2 in glycolysis and transcriptional regulators including catenin, c-Myc, HIF-1α and NF-κB, hence inhibiting cellular respiration, ATP generation and glycolysis (Tas et al., 2021).

### Inhibitors focus on lactate dehydrogenase in glycolytic pathway

LDH catalyzes the last step of glycolysis and is responsible for the mutual conversion of pyruvate and lactic acid. Natural compounds target LDH were shown in (Table 4)

**TABLE 4:** Natural compounds target lactate dehydrogenase (LDH).

Chemical Class	Compound/extract name	Types of Study	Cancer model(s)	Effects on glycolysis and potential mechanisms of action	References
Alkaloids	Matrine	*In vitro and in vivo*	Colon cancer	Inhibited mRNA and protein expression of HIF-1α and GLUT1, HK2, and LDHA expressions HCT116 and SW620 cells	Hong et al. (2019)
	Berberine	*In vitro and in vivo*	Pancreatic adenocarcinoma	Inhibited activity and protein expression of LDHA in PAAD cells	Cheng et al. (2021)
Flavonoids	Baicalein	*In vitro*	Gastric cancer	Suppressed the expression of HK2, LDHA and PDK under a hypoxic condition in AGS cells	Chen et al. (2015)
	Wogonin	*In vitro and in vivo*	Colon cancer, ovarian cancer, liver cancer	Decreased glucose uptake and PGM, HK2, GLUT1, PDHK1, and LDHA expressions in HCT116, A2780 and HepG2 cells	Zhao et al. (2018a)
		*In vitro and in vivo*	Colon Cancer	Inhibited the expression of HK2, PDK1, and LDHA in HCT116 cells and HCT116 xenograft model	Wang et al. (2014)
	Naringin	*In vitro*	Melanoma	Inhibited the expression of PKM2, LDHA, and HIF-1α in A375 cells	Guo et al. (2016)
	Quercetin	*In vitro*	Breast cancer	Inhibited glucose uptake by downregulatedthe protein levels of PKM2, GLUT1 and LDHA in MCF-7 and MDA-MB-231 cells	Jia et al. (2018), Barbosa and Martel (2020)
	Glabridin	*In vitro*	Triple-negative breast cancer	Inhibited LDH activity	Li et al. (2019d)
	Catechin	*In vitro*	Gastric cancer	Inhibited LDHA activity in SNU620/5FU cells	Han et al. (2021)
	Epigallocatecin (EGC)	*In vitro and in vivo*	Breast cancer	Decreased the expression and activity of LDHA in both MCF-7 and MDA-MB-231 cells	Wang et al. (2013b)
Non-flavonoid Phenolic Compounds	Curcumin	*In vitro*	Hepatic carcinoma	Inhibited the genes expression of HIF-1α, LDHA, MCT1, MDR1, and STAT3 in HepG2 cells	Soni et al. (2020)
	Resveratrol	*In vitro*	Colorectal cancer	Inhibited PK and LDH activity in Caco2 cells	Fouad et al. (2013), Brockmueller et al. (2021)
Terpenoids	Jolkinolide B	*In vitro and in vivo*	Melanoma	Inhibited the mRNA expression of GLUT1,GLUT3, GLUT4, HK2 and LDHA in B16F10 cells	Gao et al. (2016)
	β-escin	*In vitro and in vivo*	Ovarian Cancer	Inhibited the expression of HIF1α-targeted proteins, LDHA and HK2 in β-escin treatment omental tumors	Cheong et al. (2018), Kenny et al. (2021)
	Betulinic acid	*In vitro and in vivo*	Breast cancer	Inhibited the protein expression of c-Myc, LDHA and p-PDK1/PDK1 in MCF-7 and MDA-MB-231 cells and the mice breast cancer spontaneous model	Jiao et al. (2019)
Others	Methyl jasmonate (MJ)	*In vivo*	T Cell Lymphoma	Decreased the expression of HIF-1α, HK 2, LDHA, PDK-1, and GLUT-1	Goel et al. (2021)
	Rhizoma Paridis saponins (RPS)	*In vitro and in vivo*	Hepatocellular carcinoma	Sorafenib+RPS group remarkably Decreased the expression of FASN, CPT1, GLUT1, Myc, Akt, mTOR, and LDHA than Sorafenib group	Yao et al. (2018b)

**Alkaloids:** As demonstrated in the part of HK, matrine greatly reduces the expression of HIF-1α and its downstream regulatory targets of glucose metabolism, GLUT1, HK2 and LDHA in HCT116 and SW620 cells, which reverses the Warburg Effect (Hong et al., 2019). Cheng et al. demonstrated that berberine effectively inhibited LDHA over-expression and AMPK activation through selectively binding to LDHA, consequently inhibited cell division, migration and invasion in pancreatic cancer (Cheng et al., 2021).

**Flavonoids:** Naringin, a prominent bioflavonoid found in citrus, has been demonstrated to disrupt the c-Src/Akt signaling pathway, prevent c-Src phosphorylation and the production of downstream components PKM2, LDHA and HIF-1α. As a result, glucose metabolism was inhibited and cells growth was slowed (Guo et al., 2016). Through suppressing the expression of PKM2, GLUT1 and LDHA in MCF-7 and MDA-MB-231 cells, quercetin limited the rate of glucose absorption and lactate generation, which in turn modulated the pH of the tumor microenvironment and restricted the energy flow into tumor cells (Jia et al., 2018; Barbosa and Martel, 2020). Glabridin is a water-insoluble component derived from *Guangguo licorice*. According to a study by Li et al. (2019d), glabridin could decrease lactic acid concentrations *via* blocking LDH activity in the tumor microenvironment, which in turn blocked glycolytic metabolism in triple-negative breast cancer tumor MDA-MB-231 cells. Catechin, one of the flavonoids found in green tea, has been shown to inhibit LDHA activity, which was over-expressed in 5FU-resistant gastric cancer SNU620 cells, resulting in increased sensitivity to 5FU and apoptosis induced by ROS (Han et al., 2021). Epigallocatechin (EGC) was reported to speed up the protease degradation of HIF-1α in MCF-7 and MDA-MB-231 cells by interfering with the interaction of heat shock protein (Hsp90) with HIF-1α, according to *in vitro* and *in vivo* investigations on breast cancer by Wang et al. (2013b).

**Non-flavonoid Phenolic Compounds:** Studies have revealed that the lipophilic polyphenol curcumin has anti-cancer, anti-microbial, anti-inflammatory and anti-aging benefits (Gupta et al., 2013). A study showed that curcumin could also down-regulate glycolytic-related factors HIF-1α, LDHA, MCT1 and MDR1, STAT3 gene expression in liver cancer cells, resulting in the elevation of micro-enviromental pH to combat lactate-induced drug resistance to doxorubicin (Soni et al., 2020). Resveratrol strongly inhibited the glycolytic metabolism of colon cancer cells by decreasing the activity of glycolytic enzymes PK and LDH in colon cancer Caco2 cells (Fouad et al., 2013; Brockmueller et al., 2021).

**Terpenoids:** As demonstrated in the part of HK, JB have been demonstrated to induce cell death through the downregulation of LDHA in B16F10 cells and Dalton’s lymphoma, respectively (Gao et al., 2016). β-escin has been shown to blocked OvCa invasion (Cheong et al., 2018; Kenny et al., 2021). Betulinic acid (BA) is a naturally occurring pentacyclic terpene derived from brich bark. BA could inhibit c-myc-induced glycolytic activation and inhibited protein expression of c-Myc, LDHA, and p-PDK1/PDK1 in MCF-7 and MDA-MB-231 cells *in vitro* and mice breast cancer model suggested that BA is a good candidate for the glycolysis inhibitor *in vivo* (Jiao et al., 2019).

**Others:** MJ have been demonstrated to induce cell death through the downregulation of LDHA in B16F10 cells and Dalton’s lymphoma (Goel et al., 2021). Rhizoma paridis saponins (RPS) have been shown in previous investigations to have anti-cancer effect through modulating glycolysis and lipid metabolism (Peiyu et al., 2016). Yao et al. (2018b) confirmed that RPS with Sorafenib could lower the incidence of hepatocellular carcinoma more than Sorafenib alone. FASN, CPT1, GLUT1, Myc, Akt, mTOR and LDHA mRNA levels in liver tissue were reduced, resulting in a reduction of lactate production and inhibition of glycolysis, consequently reduced AST and ALT levels and increased AFP and MDA levels in serum, demonstrating a liver-protective role of RPS in H22 inbred mice.

### Inhibitors focus on glucose shuttling: Hypoxia-inducible factor and glucose transporters

Due to the Warburg Effect, malignant tumors have an excessively active aerobic glycolysis, necessitating a considerable amount of glucose consumption. Maintaining effective glucose transmembrane transport is therefore a crucial prerequisite for the rapid multiplication of malignant tumor cells. By modulating GLUT activity, it is possible to limit tumor cell proliferation and invasion (Brown and Wahl, 1993; Grover-McKay et al., 1998; Mueckler and Thorens, 2013; Sawayama et al., 2019). HIF-1α is a crucial component of the response to hypoxic stress. Under hypoxic conditions, HIF-1α is activated and transferred to the nucleus to bind with HIF-1β. It forms active HIF-1 and regulates the transcription of many genes including GLUT by binding with hypoxia response elements on target genes, thereby regulating the glycolytic process. Table 5 and Table 6 showed how natural compounds regulated HIF-1α and GLUT to affect glycolysis, respectively.

**TABLE 5:** Natural compounds target glucose shuttling (GLUT).

Chemical Class	Compound/extract name	Types of Study	Cancer model(s)	Effects on glycolysis and potential mechanisms of action	References
Alkaloids	Matrine	*In vitro and in vivo*	Colon cancer	Inhibited mRNA and protein expression of HIF-1α, GLUT1, HK2, and LDHA in HCT116 and SW620 cells	Hong et al. (2019)
		*In vitro and in vivo*	Colorectal cancer	Inhibited expression of GLUT1 in liver metastasis of CRC cells in mice	Li et al. (2020b)
	Tetrandrine	*In vitro*	Hepatocyte carcinoma	Downregulated the expression of GLUT1 in HepG2 cells	Yubin et al. (2019)
Flavonoids	Genistein	*In vitro and in vivo*	Hepatocellular carcinoma	Downregulated the expression of GLUT1 and HK2	Li et al. (2017a)
	Bavachinin	*In vitro and in vivo*	Cervical cancer and osteosarcoma	Decreased HIF-1 expression and VEGF, GLUT1 and HK2 expressions in HOS and KB cells	Nepal et al. (2012)
	Wogonin	*In vitro and in vivo*	Colon cancer, ovarian cancer, liver cancer	Decreased glucose uptake and PGM, HK2, GLUT1, PDHK1 and LDHA expressions in HCT116, A2780 and HepG2 cells	Zhao et al. (2018a)
	Quercetin	*In vitro*	Breast cancer	Inhibited glucose uptake by downregulatedthe protein levels of PKM2, GLUT1 and LDHA in MCF-7 and MDA-MB-231 cells	Jia et al. (2018), Barbosa and Martel (2020)
	Apigenin	*In vitro*	Pancreatic cancer	Decreased HIF-1α, GLUT1, and VEGF mRNA and protein expression in CD18 and S2-013 pancreatic cancer cells in both normoxic and hypoxic conditions	Melstrom et al. (2011)
		*In vitro*	Adenoid cystic carcinoma	Suppressed the expression of GLUT1 in a dose- and time-dependent manner in ACC-2 cells	Fang et al. (2015)
		*In vivo*	Laryngeal carcinoma	Inhibited GLUT1 expression via PI3K/Akt pathway in nude mouse model of laryngeal carcinoma	Bao et al. (2015)
		*In vitro*	Glioma	Decreased GLUT1/3, NF-κB, and PKM2 expressions in glioma stem cells	Zhao et al. (2021)
		*In vitro*	Lung carcinoma	Downregulated expression of GLUT1, GLUT3, GLUT4, PDK1 and MCT1 in EGFR mutant-resistant NSCLC cells	Chen et al. (2019)
	Phloretin	*In vitro*	Triple-negative breast cancer, liver and colon cancer	Inhibited the expression of GLUT2	Wu et al. (2009); Yang et al. (2009); Wu et al. (2018b)
	Hesperidin	*In vitro*	Breast cancer	Inhibited GLUT1 expression in MDA-MB-231 breast cancer cells	Yang et al. (2013)
	Glabridin	*In vitro*	Triple-negative breast cancer	Inhibited glucose uptake by suppressing the protein expression of GLUT1 in MDA-MB-231 cells	Li et al. (2019d)
	4-O-methylalpinumisoflavone	*In vitro*	Breast cancer	Inhibited hypoxic induction of HIF-1 target genes GLUT1 mRNA expressions in T47D cells	Liu et al. (2009)
	Kaempferol	*In vitro*	Breast cancer	Suppressed both GLUT1-mediated glucose uptake and MCT1-mediated lactate reuptake in MCF-7 cells	Azevedo et al. (2015), Martel et al. (2016)
	Epigallocatechin-3-gallate (EGCG)	*In vitro and in vivo*	Breast cancer	Decreased the expression of HIF1α and GLUT1,and also Inhibited the activity of HK, PFK, PK and LDH	Wei et al. (2018), Barbosa and Martel (2020)
Non-flavonoid Phenolic Compounds	Resveratrol	*In vitro*	Lung carcinoma	Inhibited the expression of GLUT1	Jung et al. (2013)
		*In vitro*	Leukemia	Blocked GLUT1-mediated hexose uptake in HL-60 and U-937 cell lines	Salas et al. (2013), Zambrano et al. (2019)
		*In vitro*	Breast cancer	Reduced GLUT1-mediated glucose uptake in MCF-7 breast cancer cells	Scarlatti et al. (2003), Wu et al. (2018a)
		*In vitro*	Ovarian cancer	Interruptes the GLUT1 traffic to the plasma membrane in ovarian cancer cells	Gwak et al. (2015), Zambrano et al. (2019)
	Curcumin	*In vitro and in vivo*	Lung cancer	Inhibited A549 lung cancer cells invasion and metastasis by attenuating GLUT1/MT1-MMP/MMP2 signaling	Gupta et al. (2013)
	Gossypol	*In vitro*	Leukemia	Blocks the activity of hexose transporter GLUT1 in human HL-60 cells	Perez et al. (2009)
Quinones	Emodine and Rhein	*In vitro and in vivo*	Pancreatic cancer	Inhibited HIF-1α expression and the protein levesl of its downstream targets GLUT1, HK2 and PFK-1 in MiaPaCa2 cells	Gao and Chen (2015)
Terpenoids	Oleuropein	*In vitro*	Melanoma, colon carcinoma, breast cancer, chronic myeloid leukemia.	Decreased GLUT1, MCT4 and PKM2 expressions	Ruzzolini et al. (2020)
	Jolkinolide B	*In vitro and in vivo*	Melanoma	Inhibited the mRNA expression of GLUT1, GLUT3, GLUT4, HK2 and LDHA in B16F10 cells	Li et al. (2020a)
	Prosapogenin A (PSA)	*In vitro*	Breast cancer	Inhibited the expressions of GLUT1, HK and PFKL in MCF7 cells	Cai et al. (2021), Kenny et al. (2021)
	Oridonin	*In vitro and in vivo*	Colorectal cancer	Inhibited glucose uptake and the protein expression of GLUT1 and MCT1 in all six CRC cell lines and SW480 xenograft tumors	Fujita et al. (1976), Yao et al. (2017)
Others	Graviola (annonaceous acetogenins)	*In vitro and in vivo*	Pancreatic cancer	Inhibited cellular metabolism and downregulated mRNA expression of GLUT1, GLUT4, HK2 and LDHA in PC cells	Wang et al. (2013a)
	Carpesium abrotanoides L. (CA)	*In vitro*	Breast cancer	Inhibited the expression of GLUT1, LDHA, and HK2	Goel et al. (2021)
	Aqueous extract of Kudingcha	*In vitro and in vivo*	Triple-negative breast cancer	Decreased the protein expression of GLUT1 and GLUT3 in HCC1806 and MDA-MB-231 cells	Li et al. (2016a)
	Vitamin D3	*In vitro*	Breast cancer	Inhibited the expression of GLUT1 in both MCF-7 and MDA-MB-231 cells	Li et al. (2016a)
	Melatonin	*In vitro*	Breast cancer	Decreased the protein expression of GLUT1 under acute acidosis conditions in n human breast cancer cell lines MCF-7 and MDA-MB-231	
	Momordica anti-HIV protein (MAP30)	*In vitro and in vivo*	Ovarian cancer	Reduced glucose uptake via suppressing the expression of GLUT1 and GLUT3	
	Methyl jasmonate (MJ)	*In vivo*	T Cell Lymphoma	Decreased the expression of HIF-1α, HK 2, LDHA, PDK-1, and GLUT1	Miccoli et al. (1998)
	Rhizoma Paridis saponins (RPS)	*In vitro and in vivo*	Hepatocellular carcinoma	Sorafenib+RPS group remarkably Decreased the expression of FASN, CPT1, GLUT1, Myc, Akt, mTOR, and LDHA than Sorafenib group	Yao et al. (2018b)
	β-Carotene	*In vitro and in vivo*	Neuroblastoma	Inhibited HIF-1α and its downstream targets-GLUT1 expression	Kim et al. (2014)

**TABLE 6:** Natural compounds target Hypoxia-inducible factor 1α (Hif-1α).

Chemical Class	Compound/extract name	Types of Study	Cancer model (s)	Effects on glycolysis and potential mechanisms of action	References
Alkaloids	Matrine	*in vitro and in vivo*	Colon cancer	Downregulated HIF-1α messenger RNA (mRNA) and protein expression and inhibited the expression levels of GLUT1, HK2 and LDHA in HCT116 and SW620 cells	Hong et al. (2019)
	Worenine	*in vitro*	Colon cancer	Reduced HIF-1α levels by activating p-VHL and induced down-regulation of PFK-L, HK2 and PKM2 in HCT116 and SW620 cells	Ji et al. (2021)
	Cryptolepine	*in vitro*	Breast cancer	Inhibited HIF-1 transcriptional activity and reducing hypoxia-induced HIF-1α protein expression in T47D and 4T1 cells	Zheng et al. (2022)
Flavonoids	Apigenin	*in vitro*	Pancreatic cancer	Significantly downregulated HIF-1α and GLUT-1 mRNA and protein expression in S2-013 and CD18 cells	Melstrom et al. (2011)
		*in vitro*	Glioma cancer	Downregulated the expression of GLUT-1/3, NF-κB and PKM2 by inhibited the expression of HIF-1α in GSCs cells	Zhao et al. (2021)
	Baicalein	*in vitro*	Gastric cancer	Attenuated the expression of HIF-1α by regulating the PTEN/Akt/HIF-1α signaling pathway in AGS cells	Chen et al. (2015)
		*in vitro*	Tamoxifen-resistant breast cancer	Reduced the expression of HIF-1-targeted glycolytic genes in tamoxifen-resistant breast cancer cells	Chen et al. (2021b)
	Cardamonin	*in vitro and in vivo*	Breast cancer	Inhibited the mTOR/p70S6K pathway and downregulated the expression of HIF-1α at the mRNA and protein levels in MDA-MB-231 cells	Jin et al. (2019)
	Genistein	*in vitro and in vivo*	Tamoxifen-resistant breast cancer	Limited glucose uptake, lactate production, ATP production, lactate/pyruvate ratio and expression of HIF-1α-targeted glycolytic genes Aerobic glycolysis in tamoxifen-resistant breast cancer cells	Li et al. (2017a)
	kaempferol	*in vitro*	Hepatocellular carcinoma	Inhibited MAPK and HIF-1 activities in Huh7 cells	Azevedo et al. (2015)
	Oroxylin A	*in vitro and in vivo*	Breast cancer	Inhibited the expression of HIF-1α in MDA-MB-231 cells	Wei et al. (2015)
	Silibinin	*in vitro*	Human cervical and hepatoma cells	Inhibited hypoxia-induced HIF-1alpha accumulation and HIF-1 transcriptional activity in HeLa and Hep3B cells	Garcia-Maceira and Mateo, (2009)
	Wogonin	*in vitro*	Colon Cancer	Inhibited the expression of HIF-1α and glycolysis-related proteins (HKII, PDHK1, LDHA) in HCT116 Cells	Wang et al. (2014)
Non-flavonoid Phenolic Compounds	Resveratrol	*in vitro and in vivo*	Lewis lung cancer and breast cancer	Downregulated the expression of HIF-1α and Glut-1 in HT-29 and T47D cells	Jung et al. (2013)
Quinones	Thymoquinone	*in vitro*	Renal cancer	Reduced the protein level of HIF-1α and inhibited the transcriptional activity of HIF-1α in renal cancer cells	Lee et al. (2019)
Terpenoids	Bruceine D (BD)	*in vitro and in vivo*	Hepatocellular carcinoma	Disrupted the direct interaction between β-catenin-interacting protein 1 (ICA T) and β-catenin Induced decrease in HIF-1α expression in HepG2 and Huh7 cells	Huang et al. (2021)
Others	Perillyl alcohol	*in vitro and in vivo*	Colon cancer	Inhibited HIF-1α protein synthesis, without affecting the expression level of HIF-1α mRNA or degradation of HIF-1α protein in HCT116 cells	Ma et al. (2016)
	Sulforaphane	*in vitro*	Nonmuscle invasive bladder cancer (NMIBC)	Downregulated hypoxia-induced HIF-1α and blocking HIF-1α localization to the nucleus in NMIBC cells	10.1021/acs.jafc.9b03027

**Alkaloids:** In HCT116 and SW620 cells, matrine inhibited the expression levels of downstream targets of glucose metabolism GLUT1, HK2 and LDHA by down-regulating HIF-1α mRNA and protein expression, consequently impacting tumor cell survival (Hong et al., 2019). In HCT116 and SW620 cells, wearnine decreased HIF-1 levels by activating p-VHL and triggered down-regulation of PFK-L, HK2 and PKM2, consequently limiting the glycolytic process of tumor cells and exerting anti-colorectal actions (Ji et al., 2021). Cryptolepine prevented HIF-1-mediated glycolysis and reduced the production of several glycolytic enzymes in T47D and 4T1 breast cancer cells by suppressing HIF-1 transcriptional activity and decreasing hypoxia-induced HIF-1α protein expression, resulting in decreased ATP production in tumor cells. Meanwhile, cryptolepine showed potent inhibitory effect on tumor growth in a dose-dependent manner (Zheng et al., 2022).

As described before, matrine could drastically decreased HIF-1α and its downstream regulatory targets of glucose metabolism such as GLUT1, HK2 and LDHA in HCT116 and SW620 colon cancer cells, thus reversing the Warburg Effect (Hong et al., 2019). Oxymatrine inhibited aerobic glycolysis and cancer cell invasion in colorectal cancer by suppressing the expression of PKM2 and GLUT1 in CRCs and cancer metastasis to liver in mice (Li et al., 2020b). Tetrandrine, an alkaloid extracted from the root of *Stephania tetrandra S Moore*, had been shown to have substantial impact on silicosis, autoimmune illnesses, cardiovascular diseases, and hypertension. Tetrandrine could downregulate the expression of GLUT1 in human hepatocyte carcinoma HepG2 cells, thereby decreasing the glucose uptake to cancer cells and inducing apoptosis (Yubin et al., 2019).

**Flavonoids:** In gastric cancer cells (AGS), baicalein attenuated the expression of HIF-1α by regulating the PTEN/Akt/HIF-1α signaling pathway, inhibited the process of glycolysis in tumor cells, and reversed the hypoxia-induced 5-FU drug resistance (Chen et al., 2015). Cardamonin inhibited the mTOR/p70S6K pathway and downregulated HIF-1α mRNA and protein levels in MDA-MB-231 cells, leading to increased mitochondrial oxidative phosphorylation, accumulated ROS and cell apoptosis, but decreased glucose uptake and lactic acid production (Jin et al., 2019). Oroxylin A reduced HIF-1 stability and activity in a MDA-MB-231 cells by boosting the production of the destabilizing enzyme inhibitory protein 3, consequently reducing HIF-1 expression in breast cancer cells (Wei et al., 2015). In HCT116 Cells, wogonin inhibited the expression of HIF-1α and glycolysis-related proteins (HK2, PDHK1, LDHA) by inhibiting the PI3K/Akt signaling pathway, reduced glucose uptake and lactate production to combat drug resistance of tumor cell (Wang et al., 2014). Apigenin could effectively downregulate HIF-1α and GLUT-1 mRNA and protein expression in human pancreatic cancer S2-013 and CD18 cells, and overcome any hypoxia-mediated elevation of GLUT-1 gene expression, thereby suppressing aerobic glycolysis and promoting tumor cell apoptosis (Melstrom et al., 2011). In glioma stem cells (GSCs), apigenin downregulated the expression of GLUT-1/3, NF-κB and PKM2 by inhibiting the expression of HIF-1α, inhibited the glycolysis process of cancer cells, and increased the radiosensitivity of GSCs (Zhao et al., 2021). Silibinin inhibited the mTOR/p70S6K/4E-BP1 signaling pathway and HIF-1 activity in HeLa cells and Hep3B cells under hypoxia, and reduced the rate of HIF-1α protein synthesis and inhibited aerobic glycolysis in tumor cells (Garcia-Maceira and Mateo, 2009). By increasing the interaction between PHD2 and pVHL in tamoxifen-resistant breast cancer cells, glucose uptake, lactate production, ATP production, lactate/pyruvate ratio, and expression of HIF-1-targeted glycolytic genes are all reduced. This results in a reversal of hypoxia-induced tamoxifen (TAM) tumor resistance and an increase in the antiproliferative efficacy of tamoxifen (Chen et al., 2021b). Genistein directly downregulated protein expression of HIF-1 in HCC cells, consequently inactivated GLUT1 and HK2 to impede aerobic glycolysis and accelerated tumor cell death (Li et al., 2017a). As described before, Genistein and Bavachinin, were reported to inhibited HIF-1α and GLUT1 expression in HCC, HOS and KB cells, and HK2 expression in HCC (Nepal et al., 2012; Li et al., 2017a). Wogonin has been demonstrated to decrease the expression of HK2, LDHA, and GLUT1, hence reducing glycolysis and cell proliferation in ovarian and liver cancer cells (Zhao et al., 2018a). Quercetin could reduce the acidity of the tumor microenvironment by inhibiting the glycolysis-related proteins expression of PKM2, GLUT1 and LDHA in MCF-7 and MDA-MB-231 cells, thus inhibiting glucose uptake and lactate production (Jia et al., 2018; Barbosa and Martel, 2020). Apigenin has been reported to inhibit HIF-1α, GLUT1 and VEGF mRNA and protein expression in CD18 and S2-013 cells under normoxic and hypoxic circumstances, demonstrating the anti-proliferative and anti-angiogenic effects of apigenin (Melstrom et al., 2011). Apigenin also suppressed the growth of adenoid cystic carcinoma ACC-2 cells by reducing the expression of GLUT1 (Fang et al., 2015). Bao et al. (2015) found that apigenin blocked GLUT1 expression through PI3K/Akt pathway, resulting in more sensitive to radiotherapy of laryngeal cancer in a nude mouse model. A study performed in 2021 showed that apigenin increases the radiation sensitivity of glioma stem cells by inhibiting the expression of HIF-1α, GLUT1/3, NF-κB and PKM2 in human GSCs SU3 cells and its radiation-resistant line SU3-5R (Zhao et al., 2021). It has been found that combination of apigenin and gefitinib could inhibited AMPK signaling in epidermal growth factor receptor (EGFR) resistant mutant NSCLC. The expression of c-Myc, HIF-1α and EGFR, as well as proteins related to glucose uptake like GLUT1, GLUT3, GLUT4, PDK1 and MCT1 were inhibited according to a recent study (Chen et al., 2019). Previous studies study showed that the apple polyphenol phloretin is a natural inhibitor of GLUT2, which suppressed glycolysis and tumors from spreading by inhibiting the expression of GLUT2 in MDA-MB-231 cells, liver cancer cells and colon cancer cells (Wu et al., 2009; Yang et al., 2009; Wu et al., 2018b). Hesperetin and glabridin could inhibit glucose uptake in cancer cells by decreasing the expression of GLUT1 in triple-negative MDA-MB-231 cells, consequently suppressed glycolysis in cancer cells (Yang et al., 2013; Li et al., 2019d). 4-O-methylalpinumisoflavone downregulated the expression of its downstream target genes CDKN1A, GLUT1 and VEGF in T47D cells by inactivating hypoxia-induced HIF-1α, which finally affect glycolysis and angiogenesis (Liu et al., 2009). As demonstrated in HK part, Kaempferol (Azevedo et al., 2015; Martel et al., 2016) and EGCG were significantly inhibited glycolytic pathway-related factors, such as GLUT1, HK2, PFK and LDH enzyme activities (Wei et al., 2018; Barbosa and Martel, 2020).

**Non-flavonoid Phenolic Compounds:** Resveratrol is a type of antioxidant found in grapes, peanuts, mulberries, and other foods. In lung cancer LLC cells, MCF-7, HL-60 and U-937 cells, the extracted dietary polyphenol reduced glucose uptake by reducing the expression of GLUT1 (Scarlatti et al., 2003; Jung et al., 2013; Salas et al., 2013; Zambrano et al., 2019; Brockmueller et al., 2021). Resveratrol had no effect on the expression of GLUT1 mRNA and protein, but inhibited Akt activity, preventing the transfer of intracellular GLUT1 to the plasma membrane and decreased glucose uptake in ovarian cancer cells (Gwak et al., 2015; Zambrano et al., 2019). Curcumin, a natural lipophilic polyphenol molecule derived from the roots of *Curcuma logna*, has been shown to be effective as an anti-cancer, anti-bacterial, anti-inflammatory and anti-aging agent (Gupta et al., 2013). Liao *et al.* reported reduced expression of GLUT1, MT1-MMP and MMP2 in A549 cells and suprression of lung cancer invasion and metastasis (Liao et al., 2015). Some researchers demonstrated that gossypol (a natural disesquiterpene) is a potent GLUT1 inhibitor that inhibits the activity of GLUT1 in leukemia HL-60 cells and Chinese hamster ovary (CHO) cells and disrupts the normal hexose flux (Perez et al., 2009).

**Quinones**: In renal cancer cells, thymoquinone (TQ) reduced protein level of HIF-1α through a ubiquitination-proteasome-dependent pathway and inhibited the transcriptional activity of HIF-1α. In addition, TQ altered the levels of glucose, lactate and ATP in tumor cells, leading to disturbance of anaerobic metabolism, thereby inducing apoptosis (Lee et al., 2019).

**Terpenoids:** OLEO, the main bioactive phenolic component of *Olea europaea L*, has been shown to inhibit the expression of glycolysis-related factors GLUT1, MCT4 and PKM2 in A375 cells, as well as reduced glycolysis speed, inhibiting melanoma proliferation and motility (Ruzzolini et al., 2020). Oridonin is derived from the plant *Rabdosia Rubescens*, reduces lactate output by inducing AMPK inactivation, significantly downregulates GLUT1 and MCT1 protein levels in all six CRC cell lines and SW480 xenograft tumors, inhibits glucose uptake in cancer cells resulting in metabolic imbalance, and induces autophagy (Fujita et al., 1976; Yao et al., 2017). Bruceine D (BD) inhibited HIF-1α-mediated metabolism in HepG2 and Huh7 cells by inducing β-catenin degradation *via* directly disrupting contact between β-catenin-interacting protein 1 and β-catenin (Huang et al., 2021).

**Others:** Highly abundant in dark green or orange fruits and vegetables, β-carotene is an active vitamin A precursor. β-carotene can considerably suppress the expression of HIF-1α and its downstream targets, VEGF and GLUT1, in malignant SK-N-BE(2) C neuroblastoma cells and their liver metastases, thereby limiting the invasion and migration of human neuroblastoma cells (Kim et al., 2014). Perillyl alcohol (POH) suppressed the mTOR/4E-BP1 signaling pathway in HCT116, HeLa and SK-Hep1 cells, but had no effect on the HIF-1 mRNA level or HIF-1 protein degradation. Additionally, POH decreased expression of cyclins D1, c-Myc and Skp2 and elevated expression of p53 and p21, which caused cell cycle arrest in the G1 phase (Ma et al., 2016).

### Inhibitors focus on lactate shuttling

Cancer cells metabolize glucose through anaerobic glycolysis, and the final product is lactic acid formed in the cytoplasm. To avoid intercellular acidification, excess lactic acid and protons are excreted by monocarboxylic acid transporters (MCTs), which are often overdose in different malignancies. Targeted MCT-mediated lactate/proton outflow makes MCT a potential anti-cancer target. Natural compounds targets MCTs were shown in Table 7 as bellow.

**TABLE 7:** Natural compounds target Monocarboxylic acid transporter (MCT)

Chemical Class	Compound/extract name	Types of Study	Cancer model(s)	Effects on glycolysis and potential mechanisms of action	References
Flavonoids	Kaempferol	*In vitro*	Breast cancer	Suppressed both GLUT1-mediated glucose uptake and MCT1-mediated lactate reuptake in MCF-7 cells	Azevedo et al. (2015), Martel et al. (2016)
	Apigenin	*In vitro*	Lung carcinoma	Downregulated expression of GLUT1, GLUT3, GLUT4, PDK1 and MCT1 in EGFR mutant-resistant NSCLC cells	Chen et al. (2019)
	Wogonin	*In vitro*	Gastric cancer, Lung adenocarcinoma	Decreased HIF-1α and MCT-4 expression in SCG-7901 cells	Tai et al. (2005), Wang et al. (2019)
	Quercetin	*in vitro*	Breast cancer	Inhibited MCT expression	Morais-Santos et al. (2014), Martel et al. (2016)
Non-flavonoid Phenolic Compounds	Curcumin	*In vitro*	Hepatic carcinoma	Inhibited genes expression of HIF-1α, LDHA, MCT1, MDR1 and STAT3 in HepG2 cells	Soni et al. (2020)
Terpenoids	Oleuropein	*In vitro*	Melanoma, colon carcinoma, breast cancer and chronic myeloid leukemia	Decreased GLUT1, MCT4 and PKM2 expressions	Ruzzolini et al. (2020)
	Oridonin	*In vitro and in vivo*	Colorectal cancer	Inhibited glucose uptake and the protein expression of GLUT1 and MCT1 in all six CRC cell lines and SW480 xenograft tumors	Fujita et al. (1976), Yao et al. (2017)

To test whether flavonoid polyphenol wogonin can stop gastric cancer cells from multiplying *in vitro*, researchers used SGC-7901 cells treated with flavonoid polyphenol wogonin, as a result, HIF-1α and MCT-4 expression was inhibit, therefore cell proliferation and pathogenesis were inhibited (Tai et al., 2005; Wang et al., 2019). Polyhydroxy flavonoid, quercetin is typically found in the form of glycosides when it is paired with a sugar. Natural flavonoid kaempferol can be found in a wide variety of fruits and vegetables. Quercetin has been demonstrated to interfere with MCT1 mediated lactate transfer in breast cancer by reducing MCT1 expression (Morais-Santos et al., 2014; Martel et al., 2016). GLUT1-mediated glucose uptake and extracellular transport of lactic acid are both inhibited by kaempferol in breast cancer cells (Azevedo et al., 2015; Martel et al., 2016). In addition, the antioxidant, antiviral, antibacterial, and anti-inflammatory properties of quercetin and kaempferol have been discovered (Azevedo et al., 2015; Martel et al., 2016).

Terpenoids are a class of organic hydrocarbons that are found in large quantities in plants and have potent anticancer properties. Oleuropein is a member of the secoiridoid glycosides class. It has a potent antioxidant impact and is the most active natural component taken from olive leaves. Oleuropein was demonstrated to be able to prevent the development of melanoma, colorectal, breast, and chronic myoid leukemia by suppressing the expression of GLUT1, MCT1 and PKM2 in the glycolysis pathway (Ruzzolini et al., 2020). Oridonin has potent anticancer properties by suppressing tumor cell proliferation, activating autophagy, and causing cell death (Fujita et al., 1976; Yao et al., 2017).

## Conclusion and perspectives

### Mechanisms

Studies have demonstrated that alkaloids have anticancer effects through slowing glycolysis, in addition to their well-known antimicrobial, antiarrhythmic, antihypertensive and lipid-regulating properties. All three compounds, Berberine, Sinomenine and NK007, inhibit HK2-mediated glycolysis to varying degrees, leading to apoptosis induction and chemosensitization, Berberine is a functional inhibitor of LDHA. Tetrandrine decreases GLUT1 expression and in turn decreases cancer cell proliferation. Dauricine, matrine and oxymatrine are all glucose nutrients that inhibit aerobic glycolysis and cancer cell invasion by interacting with HK2, PKM2, GLUT1, LDHA or MCT in varying degrees.

Experiments conducted *in vitro* and *in vivo* indicated that flavonoids in polyphenolic natural products prevent lung cancer, stomach cancer, breast cancer and hepatocellular carcinoma. According to studies, the anticancer impact of polyphenols is correlated with the inhibition of glycolysis in cancer cells. Chrysin, oroxylin A and icariin exert the majority of their actions by reducing the expression of HK2 in cancer cells, whereas glabridin decreases the quantity of lactic acid (LD) in the tumor microenvironment by blocking LDH activity in cancer cells. Inhibiting the expression of GLUT1 gives Apigenin, hesperetin and glabridin key antitumor capabilities in anti-cell proliferation and radio-chemosensitization. In addition, the action of several polyphenolic natural compounds is multi-target, such as baicalin, wogonin, quercetin and apigenin, which inhibit glycolysis to varied degrees by acting synergistically on HK2, PDK, GLUT1, LDHA or MCT.

Non-flavonoid Phenolic Compounds, an important group of natural polyphenols, can also stop glycolysis and cause lung, ovarian, and breast cancer cells to die by apoptosis. Xanthohumol, EGCG and resveratrol mostly work by stopping cancer cells from making HK2. Catechin, EGCG and resveratrol speed up apoptosis by stopping cancer cells from making LDHA. Resveratrol can not only stop GLUT1 from being made, but it can also stop intracellular GLUT1 from moving to the plasma membrane and stop glucose from being taken in.

To a large extent, terpenoids such alcohols, aldehydes, ketones, carboxylic acids, esters and glycosides are responsible for blocking the glycolysis of malignant cells like melanoma and neuroblastoma. Inhibiting HK2 expression in cancer cells is possible with dioscin, NA and TPL, and decreasing HK2 activity is possible with limonin by increasing HK2 translocation from mitochondria to the cytoplasm. This results in energy crisis and apoptosis in cancer cells. Gossypol and β-carotene are likewise potent GLUT1 inhibitors that suppress GLUT1 function and interfere with normal glucose metabolism. It was also discovered that small amounts of JB, MJ, OA, oleuropein, PSA, BA, RPS, oridoine and β-carotene were present. By inhibiting GLUT1, LDHA or MCT, or activating HK2, the Warburg Effect was countered.

Tanshinone IIA, a quinone, has been demonstrated to suppress HK2 expression in cancer cells. Emodin and rhein inhibit cancer cells *via* the Warburg Effect by downregulating GLUT1, HK2 and PFK1 expression.

Other natural products, such as deguelin, reduced the expression or activity of HK2, thus inhibiting glycolysis and causing apoptosis. Vitamin D3 and melatonin stops tumors from spreading by inhibiting the expression of GLUT1 in cancer cells with an aggressive phenotype. Graviola, CA, MED and kudingcha each reduced the expression of key glycolysis enzymes HK2, PKM2, GLUT1, GLUT3 and LDHA to different degrees, which stopped cancer cells from growing and migration.

### Investigation of clinical applicability

Chinese herbal medicine has been utilized to treat a variety of diseases, particularly cancer for thousands of years, and the effective substances have always been the focus of research (Wang et al., 2012b). Natural products have less side effects than radiotherapy and chemotherapy and are well tolerated by the majority of patients (Wang and Sun, 1995). Flavonoids are a type of natural polyphenol that has been found to have anticancer effects *via* altering ROS-scavenging enzyme activities, halting cell cycle, causing apoptosis, autophagy and reducing cell proliferation and invasiveness (Kopustinskiene et al., 2020). Furthermore, the Chinese herbal medication Curcuma wenyujin has piqued the interest of numerous researchers as an anti-tumor agent. As a second-line anti-tumor medicine that has been clinically used in China for more than 20 years, its principal effective constituent, β-elemene, displays clinical anti-tumor activity through several modes of action, with potential clinical and scientific relevance (Bai et al., 2021).

Chinese herbal remedies are rich in primary and secondary metabolites that deal with biological and structural variety. These natural compounds not only play a crucial function in organism defense and physiological regulation, but they also contribute significantly to human health. At the moment, natural products are mostly derived from plants, animals, microbes, and marine species, which are the primary sources of leading chemicals and new medications. Nature’s products will play a vital part in the development of new revolutionary medications, insecticides, cell cycle, information transmission and intracellular protein delivery research in the future.

## Highlights

This article examined the most recent research on the anti-cancer effect of natural compounds *via* glycolysis. The results are clearly presented in table format. The relationship between natural products and the glycolysis process is examined in further detail in this paper, beginning with the target of the glycolysis pathway. It is new in this paper to focus on the structure of natural compounds and their anti-cancer properties.

## Glossary

AP: Apigenin
BA: Betulinic acid
BD: Bruceine D
CA: Carpesium abrotanoides L.
CK: Compound K
CYN: Cynaropicrin
Dau: Dauricine
DHA: Dihydroartemisinin
EGC: Epigallocatechin
EGCG: Epigallocatechin-3-gallate
IVT: Isovitexin
JB: Jolkinolide B
MCL: Micheliolide
MED: Mycoepoxydiene
MJ: Methyl jasmonate
OA: Oleanolic acid
PHY: Physciosporin
POH: Perillyl alcohol
PSA: Prosapogenin A
RPS: Rhizoma paridis saponins
TA: Tannins acid
TAM: Tamoxifen
TPL: Triptolide
TQ: Thymoquinone
UA: Ursolic acid
ADP: Adenosine diphosphate
ATP: Adenosine triphosphate
EGFR: Epidermal growth factor receptor
GLUT: Glucose transporters
HIF: Hypoxia-inducible factor
HK: Hexokinase
LD: Lactic acid
LDH: Lactate dehydrogenase
MCTs: Monocarboxylic acid transporters
MMP: Matrix metalloproteinase
PFK1: Phosphofructokinase 1
PFK-1 Phosphofructokinase-1
PFKFB3: Phosphofructokinase-2/fructose-2,6-diphosphate 3
PI: Pnorganic phosphate
PK: Pyruvate kinase
VDAC: Voltage-dependent anion channels
VEGF: Vndothelial growth factor
