# Supplementary material for: Natural products targeting glycolytic signaling pathways-an updated review on anti-cancer therapy
Source: Front Pharmacol. 2022 Oct 20;13:1035882. doi: 10.3389/fphar.2022.1035882 (PMC9631946; doi:10.3389/fphar.2022.1035882)
Supplement: Supplementary file 1 [file DataSheet1.docx]

Supplementary data

**Figure 1. Cell lines used in the study**

| **Cell line** | **Abbreviations** | **Origins** | **Cancer models** |
| --- | --- | --- | --- |
| Human ovarian cancer cell line | SKOV3 | Ascitic fluid of ovarian adenocarcinoma; epithelioid cell | Ovarian adenocarcinoma |
| Human ovarian cancer cell line | 3AO | Ovary; epithelioid cell | Ovarian adenocarcinoma |
| Human hepatocellular carcinoma cell line | HEPG2 | Liver tissue; epithelioid cell | Liver cancer |
| Human high metastatic hepatocellular carcinoma cell line | HCC-LM3 | Liver tissue; epithelioid cell | Liver cancer |
| Human high metastatic hepatocellular carcinoma cell line | LM3 | Liver tissue; epithelioid cell | Liver cancer |
| Human hepatocellular carcinoma cell line | SMMC-7721 | Liver tissue; epithelioid cell | Liver cancer |
| Human hepatocellular carcinoma cell line | Hep3B | Liver tissue; epithelioid cell | Liver cancer |
| Human hepatocellular carcinoma cell line | Huh-7 | Liver tissue; epithelioid cell | Liver cancer |
| Human hepatocellular carcinoma cell line | SK-Hep1 | Ascitic fluid of liver adenocarcinoma; endothelioid cell | Liver cancer |
| Mouse hepatocellular carcinoma cell line | H22 | Ascitic fluid of mouse hepatocarcinoma; Lymphoblastoid cell | Liver cancer |
| Human colorectal carcinoma cell line | HCT116 | Colon tissue; epithelioid cell | Colorectal cancer |
| Human colorectal carcinoma cell line | SW-620 | Large intestine tissue; epithelioid cell | Colorectal adenocarcinoma |
| Human colorectal carcinoma cell line | HT-29 | Primary colon tumor tissue; epithelioid cell | Colorectal adenocarcinoma |
| Human colorectal carcinoma cell line | Caco2 | Colon tissue; epithelioid cell | Colorectal adenocarcinoma |
| Human colorectal carcinoma cell line | SW480 | Large intestine tissue; epithelioid cell | Colorectal adenocarcinoma |
| Human colorectal carcinoma cell line | DLD-1 | Large intestine tissue; epithelioid cell | Colorectal adenocarcinoma |
| Human gastric adenocarcinoma cell line | AGS | Stomach tissue; epithelioid cell | Gastric adenocarcinoma |
| Human gastric cancer cell line | MKN-45 | Lymphonode gastrici in patients with signet-ring cell carcinoma | Gastric cancer |
| Human gastric cancer cell line | SGC-7901 | Lymph node metastasis of gastric adenocarcinoma; epithelioid cell | Gastric adenocarcinoma |
| Human gastric cancer cell line | SNU620 | Stomach tissue; epithelioid cell | Gastric cancer |
| Human breast cancer cell line | MDA-MB-231 | Pleural effusion cells of metastatic breast adenocarcinoma; epithelioid cell | Breast adenocarcinoma |
| Human breast cancer cell line | MCF7 | Breast tissue; epithelioid cell | Breast adenocarcinoma |
| Human breast cancer cell line | SK-BR-3 | Pleural effusion cells of breast adenocarcinoma; epithelioid cell | Breast adenocarcinoma |
| Human breast ductal carcinoma cell line | T47D | Pleural effusion cells of breast ductal carcinoma; epithelioid cell | Breast ductal carcinoma |
| Mouse breast cancer cell line | 4T1 | Breast tissue; epithelioid cell | Breast cancer |
| Human glioma cell line | U87 | Brain malignant gliomas; epithelioid cell | Glioblastoma |
| Human glioma cell line | SU3 | Glioblastoma multiforme; adopted from the Department of Neurosurgery of the Second Afliated Hospital of Soochow University (Suzhou, China) | Glioblastoma |
| Human glioblastoma cell line | T98-G | Brain tissue; Fibroblast-like cell | Glioblastoma multiforme |
| Human neuroblastoma cell line | SK-N-BE(2)C | Bone marrow aspirate from patients with neuroblastoma | Neuroblastoma |
| Human pancreatic cancer cell line | SW 1990 | Spleen metastasis of pancreatic adenocarcinoma; epithelioid cell | Pancreatic adenocarcinoma |
| Human pancreatic cancer cell line | MiaPaCa2 | Pancreas tissue; epithelioid cell | Pancreatic cancer |
| Human metastatic pancreatic adenocarcinoma cell line | AsPC-1 | Nude mouse xenografts initiated with cells from the ascites of a patient with pancreatic adenocarcinoma | Pancreatic adenocarcinoma |
| Human pancreatic cancer cell line | BxPC-3 | Pancreas tissue; epithelioid cell | Pancreatic adenocarcinoma |
| Human pancreatic cancer cell line | Mia PaCa-2 | Pancreas tissue; epithelioid cell | Pancreatic cancer |
| Human pancreatic cancer cell line | CD18 | Peritoneal ascitic fluid of primary pancreatic adenocarcinoma and metastases to the liver, diaphragm and lymph nodes; epithelioid cell | Pancreatic adenocarcinoma |
| Human pancreatic cancer cell line | S2-013 | Subline of the human pancreatic cancer cell line  (SUIT-2); adopted from Dr. Takeshi Iwamura (Japan) | Pancreatic cancer |
| Human prostatic cancer cell line | PC3 | Bone metastasis of prostatic adenocarcinoma; epithelioid cell | Prostatic adenocarcinoma |
| Human prostatic cancer cell line | DU145 | Brain tissue; epithelioid cell | Prostate cancer |
| Human diffuse large B-cell lymphoma cell line | OCI-Ly1 | Peripheral blood | Diffuse large B-cell lymphoma |
| Human diffuse large B-cell lymphoma cell line | OCI-Ly18 | Peripheral blood | Diffuse large B-cell lymphoma |
| Human lung cancer cell line | A549 | Alveolar basal epithelial cell | Non-small cell lung cancer |
| Human lung cancer cell line | H1975 | Lung tissue; epithelioid cell | Non-small cell lung cancer |
| Human lung cancer cell line | H446 | Pleural fluid of small cell cancer of the lung; epithelioid cell | Lung carcinoma |
| Human lung cancer cell line | H1299 | Lung tissue; Epithelial-like cell | Non-small cell lung cancer |
| Human lung cancer cell line | HCC827 | Lung tissue; epithelioid cell | Non-small cell lung cancer |
| Human lung cancer cell line | PC9 | Lung tissue; epithelioid cell | Non-small cell lung cancer |
| Mouse lung cancer cell line | LLC | Lung tissue | Lewis lung carcinoma |
| Human cervical cancer cell line | HeLa | Cervix tissue; epithelioid cell | Cervical adenocarcinoma |
| Human pharyngeal squamous cell carcinoma cell line | FaDu | hypopharyngeal tumor; epithelioid cell | Pharyngeal cancer (squamous cell carcinoma) |
| Pleural effusion of metastatic Pharyngeal cancer | Detroit 562 | Pharynx tissue; epithelioid cell | Pharyngeal cancer |
| Human tongue squamous cell carcinoma | Cal27 | Tongue tissue taken prior to treatment; epithelioid cell | Pharyngeal cancer (squamous cell carcinoma) |
| Human malignant melanoma cell line | A375 | Skin tissue; epithelioid cell | Malignant melanoma |
| Mouse melanoma cell line | B16 | Skin tissue; fibroblast-like cell | Melanoma |
| Mouse melanoma cell line | B16F10 | Skin tissue; epithelioid cell | Melanoma |
| Human melanoma cell line | MEL526 | Melanoma cells | Melanoma |
| Human melanoma cell line | MEL697 | Melanoma cells | Melanoma |
| Human melanoma cell line | MEL103 | Melanoma cells | Melanoma |
| Mouse epidermal cell line | JB6 | Skin tissue; epithelioid cell | Normal |
| Human esophageal cancer cell line | Ec109 | The middle esophageal of squamous cell carcinoma; epithelioid cell | Esophageal cancer |
| Human esophageal cancer cell line | Eca109 | The middle esophageal of squamous cell carcinoma; epithelioid cell | Esophageal cancer |
| Human esophageal cancer cell line | Ec9706 | Esophagus tissue; epithelioid cell | Esophageal cancer |
| Human promyeloid leukemia cell line | HL-60 | Peripheral blood; Promyeoloblast; Lymphoblast-like cell | Acute promyelocytic leukemia |
| Human salivary adenoid cystic carcinoma cell line | ACC-2 | Salivary adenoid cystic carcinoma tissue; epithelioid cell | Salivary adenoid cystic carcinoma |
| Human histiocytic lymphoma cell line | U-937 | Pleural effusion of  histiocytic lymphoma; monocyte | Histiocytic lymphoma |
| Human osteosarcoma cell line | HOS | Bone tissue; Mixture of fibroblast and epithelial like cells | Osteosarcoma |
| Human oral cancer cell line | KB | Shown to be Hela derivative; epithelioid cell | Oral cancer (papilloma) |
